# Magnetoliposomes as Contrast Agents for Longitudinal *in vivo* Assessment of Transplanted Pancreatic Islets in a Diabetic Rat Model

**DOI:** 10.1038/s41598-018-29136-9

**Published:** 2018-07-31

**Authors:** Rita Sofia Garcia Ribeiro, Conny Gysemans, João Paulo Monteiro Carvalho Mori da Cunha, Bella B. Manshian, Daniel Jirak, Jan Kriz, Juan Gallo, Manuel Bañobre-López, Tom Struys, Marcel De Cuyper, Chantal Mathieu, Stefaan J. Soenen, Willy Gsell, Uwe Himmelreich

**Affiliations:** 1 Biomedical MRI/MoSAIC, Department of Imaging and Pathology, Biomedical Sciences Group, KU LEUVEN, Herestraat 49, 3000 Leuven, Belgium; 2 Clinical and Experimental Endocrinology, Department of Chronic Diseases, Metabolism and Ageing, KU LEUVEN, Herestraat 49, 3000 Leuven, Belgium; 3 MR Spectroscopy Unit, Institute for Clinical and Experimental Medicine (IKEM), Videnska 1958/9, 140 21, Prague, Czech Republic; 4 Department of Biophysics, Institute of Biophysics and Informatics, First Faculty of Medicine, Charles University, Salmovska 1, 120 00, Prague 2, Czech Republic; 5 Diabetes Center, Institute for Clinical and Experimental Medicine (IKEM), Videnska 1958/9, 140 21, Prague, Czech Republic; 6 Diagnostic Tools & Methods/Advanced (magnetic) Theranostic Nanostructures Lab, International Iberian Nanotechnology Laboratory (INL), Av. Mestre José Veiga s/n, 4715-330 Braga, Portugal; 7 Lab of Histology, Biomedical Research Institute, Hasselt University, Campus Diepenbeek, Agoralaan, B3590 Diepenbeek, Belgium; 8 Laboratory of BioNanoColloids, Interdisciplinary Research Centre, KULAK/KU LEUVEN, Etienne Sabbelaan 53, 8500 Kortrijk, Belgium

## Abstract

Magnetoliposomes (MLs) were synthesized and tested for longitudinal monitoring of transplanted pancreatic islets using magnetic resonance imaging (MRI) in rat models. The rat insulinoma cell line INS-1E and isolated pancreatic islets from outbred and inbred rats were used to optimize labeling conditions *in vitro*. Strong MRI contrast was generated by islets exposed to 50 µg Fe/ml for 24 hours without any increased cell death, loss of function or other signs of toxicity. *In vivo* experiments showed that pancreatic islets (50–1000 units) labeled with MLs were detectable for up to 6 weeks post-transplantation in the kidney subcapsular space. Islets were also monitored for two weeks following transplantation through the portal vein of the liver. Hereby, islets labeled with MLs and transplanted under the left kidney capsule were able to correct hyperglycemia and had stable MRI signals until nephrectomy. Interestingly, *in vivo* MRI of streptozotocin induced diabetic rats transplanted with allogeneic islets demonstrated loss of MRI contrast between 7–16 days, indicative of loss of islet structure. MLs used in this study were not only beneficial for monitoring the location of transplanted islets *in vivo* with high sensitivity but also reported on islet integrity and hereby indirectly on islet function and rejection.

## Introduction

Type 1 diabetes (T1D) is a chronic autoimmune disease caused by the selective destruction of the insulin-producing beta-cells in the pancreatic islets of Langerhans, resulting in insulin deficiency and hyperglycaemia^1,2^. T1D patients depend on exogenous insulin therapy for survival^3,4^.

A potential alternative treatment of T1D, in particular in patients that are inadequately regulated by insulin injection and experience severe hypoglycaemia, is the transplantation of pancreatic islets^4–6^. Despite promising results short-term in clinical trials, with over 50% of subjects reaching insulin independence at one year post engraftment, insulin independence is usually not sustainable in the long-term, with 85–90% of these patients requiring insulin injections by five years post-transplantation^7,8^. The failure of long-term insulin independence may be attributed to islet loss at the transplantation site due to factors like the transplantation procedure^9,10^, progressive immune rejection^11,12^, toxicity due to continued use of immunosuppressive drugs^13,14^ or ischemia due to hypoxia from the initial lack of islet vascularization^15–17^. Therefore, there is a strong need for real-time assessment of functional islet grafts as the majority of islet injury in clinical transplantation takes place before apparent changes in recipient’s glycaemic levels. For repeated assessment, procedures should be as non-invasive as possible. Magnetic resonance imaging (MRI) of islets labelled with superparamagnetic iron oxide nanoparticles (SPIO) is one promising solution. Several commercially used and FDA-approved SPIOs (such as ferumoxides and ferucarbotran), have already been tested in several clinical^18–20^ and preclinical^21–23^ studies in combination with MRI, showing the ability to track the location and integrity of transplanted islets non-invasively and longitudinally.

However, most of the FDA-approved SPIOs, which were used in cell tracking studies^24–26^, were not designed for cell labelling and therefore not optimally suited due to the relatively low intrinsic uptake efficiencies. In addition, cell labelling by using SPIOs often requires transfection agents^27^ Some of those particles were also withdrawn from the market due to economic reasons^28^. Most of the FDA-approved SPIOs are dextran coated (*e*.*g*. Endorem® in the EU, Feridex® in the US). Dextran is a large macromolecule, which continually undergoes conformational changes and is even found to completely desorb from the particle surface. This can rapidly result in bare iron oxide cores exposed to the degradative lysosomal environment, causing oxidative stress and loss of MRI contrast^29^.

Consequently, alternative contrast agents for applications in clinical cell therapy, including islet transplantation, are urgently required. One alternative is the use of phospholipid-coated ultra-small SPIOs called magnetoliposomes (MLs)^30^. MLs consist of a phospholipid bilayer with an inner layer strongly chemisorbed onto the iron oxide core, while an outer layer is more loosely adsorbed allowing easy modifications of this outer layer transferring functionalized lipids between MLs and pre-functionalized liposomes^30^. These MLs have limited toxicity, result in relatively rapid cellular uptake and allow high intracellular iron concentrations in a wide variety of cell types, including mesenchymal stem cells, immune and others^31^. Using well-tolerated concentrations of MLs and Endorem® for labelling stem cells showed superior imaging properties of MLs for *in vivo* cell visualization. These data indicate that MLs are better suited for *in vivo* MRI of pre-labelled, grafted cells than Endorem® if comparable iron concentrations are used^32^.

The goals of this study were to assess the suitability of MLs for islet labelling and optimize labelling conditions with the aim to minimize exposure concentrations in beta-cell like cell lines and pancreatic islets *in vitro* and validate the ability to visualize islets labelled with MLs *in vivo* by MRI. Furthermore, it is crucial for the outcome of transplantation that pancreatic islets labelled with an MRI probe retain their full functionality, in particular their ability to secrete insulin. Therefore, we put particular emphasis on whether islets labelled with MLs will restore normoglycaemia in diabetic rats.

## Results

### Characterization of MLs

**Figure 1:**
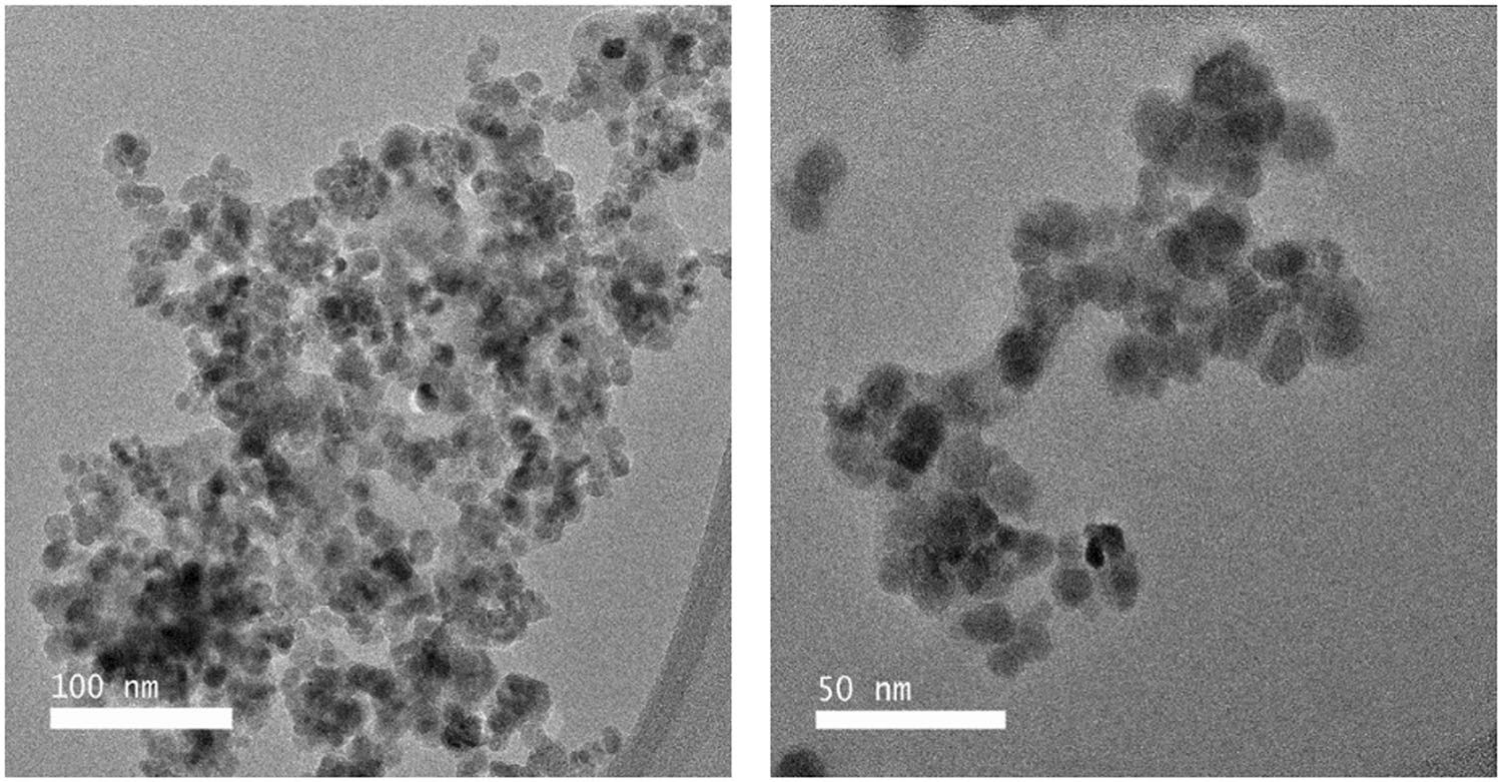
TEM image of anionic MLs. (Note the light shell around the magnetite particles, indicating the presence of the phospholipid bilayer.) (**A**) Scale bar = 100 nm; (**B**) Scale bar = 50 nm.

MLs were synthesized as described^33^. They were characterized by TEM, DLS, zeta potential and relaxivity (r_1_/r_2_) measurements. TEM indicates a spherical morphology of iron oxide particles, each individually enveloped by a phospholipid bilayer (Fig. 1). A phospholipid/Fe_3_O_4_ (mmol/g) ratio of 7.56 ± 0.06 was calculated, indicating an intact phospholipidic bilayer. DLS analysis revealed a hydrodynamic diameter of 53.5 ± 0.3 nm with a polydispersity index (PDI) of 0.36 ± 0.07. The zeta potential was −59.75 ± 5.12 mV. The relaxivity of anionic MLs (351 ± 3 s^−1^/mM) is about two times higher than that of other SPIO particles, e.g. 151 ± 6 s^−1^/mM or Resovist® at the same temperature and field strength. A summary of the physico-chemical properties of anionic MLs in comparison with SPIOs previously used to label pancreatic islets is provided in Table S1.

### Optimization of cell labelling and uptake confirmation in rat INS-1E cells and pancreatic islets

**Figure 2:**
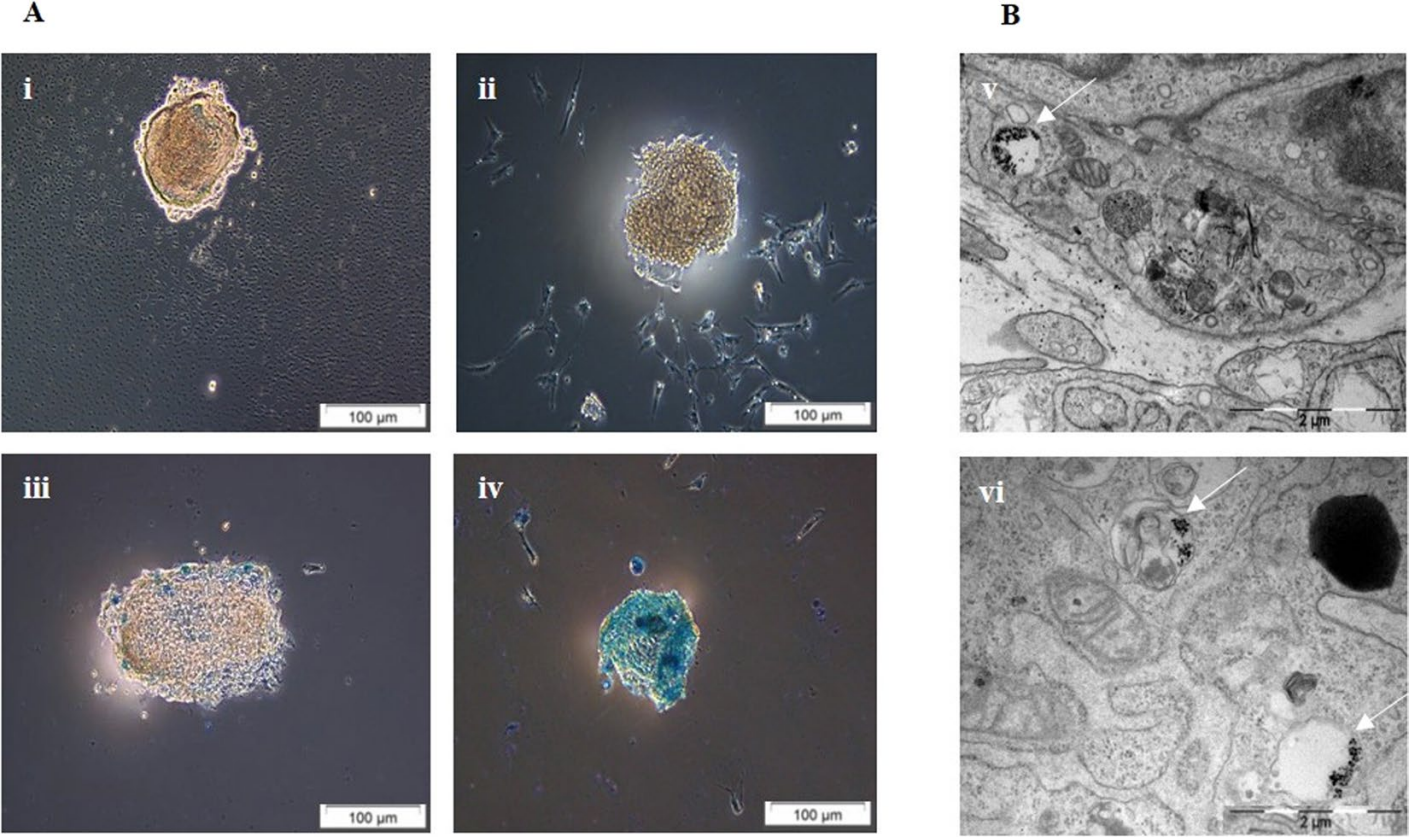
(**A**) Iron uptake by rat islets was confirmed by Prussian blue staining (i) control, (ii) 10 µg Fe/mL, (iii) 25 µg Fe/mL and (iv) 50 µg Fe/mL. Scale bar: 100 µm (**B**) TEM of (v, vi) ML-labelled islets confirms the presence of particles in endosomal structures of cells (as indicated by white arrows). Scale bar: 2 µm.

The rat insulinoma cell line INS-1E (*n* = 1500) was labelled *in vitro* with anionic MLs at increasing concentrations of MLs (5–100 µg Fe/ml). Optimal MRI contrast without any significant effect on viability, oxidative stress or cell morphology was achieved in cells when labelled with 50 µg Fe/ml of MLs for 24 hrs (ESM Fig. 1). To test the MR detectability of cells labelled under different conditions, agarose phantoms of pancreatic islets (*n* = 100) labelled with 0, 10, 25 and 50 µg Fe/ml were prepared, and MRI was performed. The T2/T2* relaxivity of labelled islets decreased compared to non-labelled islets, 50.8 ± 16.2/14.5 + 3.5 ms in comparison with 73.0 ± 13.2/39.7 ± 12.5 ms for non-labelled islets (Table S2). We also obtained a linear correlation between relaxation time and the intracellular iron concentration (r^2^ = 0.98), quantified by inductively coupled plasma optical emission spectrometry (ICP-OES), at a labelling concentration of 50 µg Fe/ml, which resulted in 189 ± 0.84 ng Fe/islet (Figure S2). Prussian blue staining and TEM further supported internalization of MLs into islet cells (Fig. 2).

### *In vitro* assessment of islet viability and function

**Figure 3:**
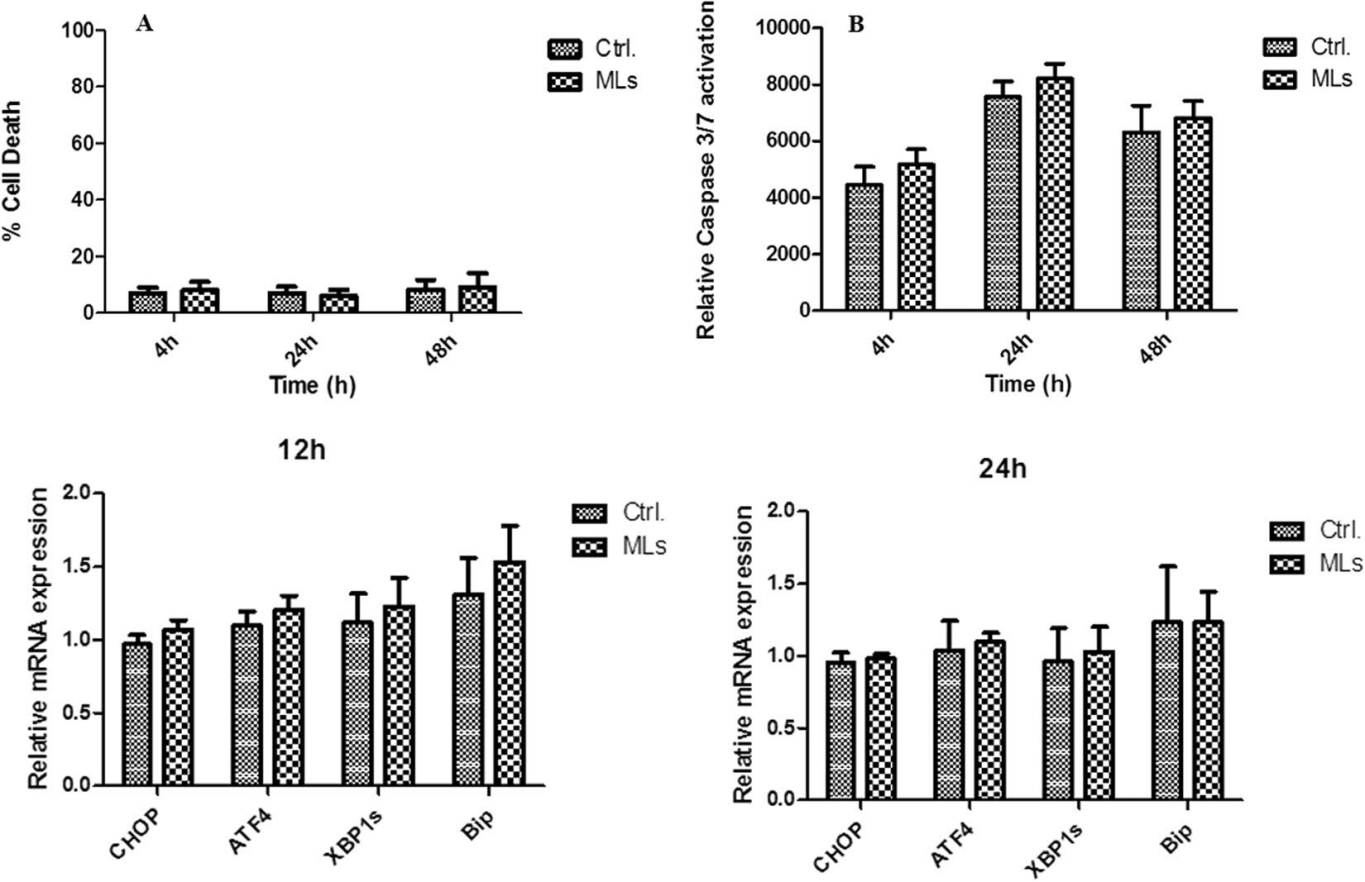
(**A**) Cell death rate was determined with Hoechst-PI staining for INS-1E cells after 4, 24 and 48 hrs of exposure to MLs (50 µg Fe/mL) (**B**) Caspase 3/7 activation in INS-1E cells after 4, 24 and 48 hrs of exposure to MLs (50 µg Fe/mL). One unit caspase (0.07 ng protein) is the amount of enzyme required to cleave 1 pmol of substrate (Ac-DEVD-pNA) hydrolysed/minute at 30 °C according to the manufacturer’s unit definition. There was no statistically significant difference between labelled islets and unlabelled controls. Data are presented as mean ± SEM (n = 9). **(C**,**D)** mRNA levels of ER stress mediators, CCAAT/-enhancer-binding protein homologous protein (Chop, n = 5), Activating Transcription Factor 4 (ATF4, n = 5), binding immunoglobulin protein (Bip, n = 5) and spliced X-box binding protein (Xbp1s, n = 5), are not up-regulated in INS-1E cells after 12 and 24 hrs, respectively, of exposure to MLs. There was no statistically significant difference between labelled islets vs. control. Data are presented as mean ± SEM vs. control (housekeeping genes).

We evaluated whether the viability of INS1-E cells was affected after labelling with MLs for different time intervals (4, 24 and 48 hrs). Even after 48 hrs of exposure to MLs (50 µg Fe/ml), INS-1E cells were viable with no measurable effect on cell survival, with 8% of dead cells after ML labelling compared to 9% cell death under control conditions (Fig. 3A). There was also no significant activation of caspase 3/7 pathways after exposure to MLs for 4, 24 and 48 hrs in INS-1E cells, with an 13.8, 8.2 and 7.3% increase in relative caspase activation compared to controls (P < 0.05), suggesting minimal apoptosis in single beta-cells after labelling with MLs (Fig. 3B). Additionally, mRNA levels of CHOP, ATF4, Bip and of Xbp1s did not increase following 24 hrs of labelling with MLs (Fig. 3C,D), confirming that there was no induction of endoplasmic reticulum (ER) stress, which often precedes apoptotic cell death. Glucose-stimulated insulin release was expressed as the stimulation index, calculated as the ratio of stimulated (30 mM) to basal (3 mM) insulin release (Figure S3). No differences in insulin secretion between unlabeled and labeled islets were found.

### *In vivo* and *ex vivo* follow up of transplanted islets labelled with MLs by MRI

**Figure 4:**
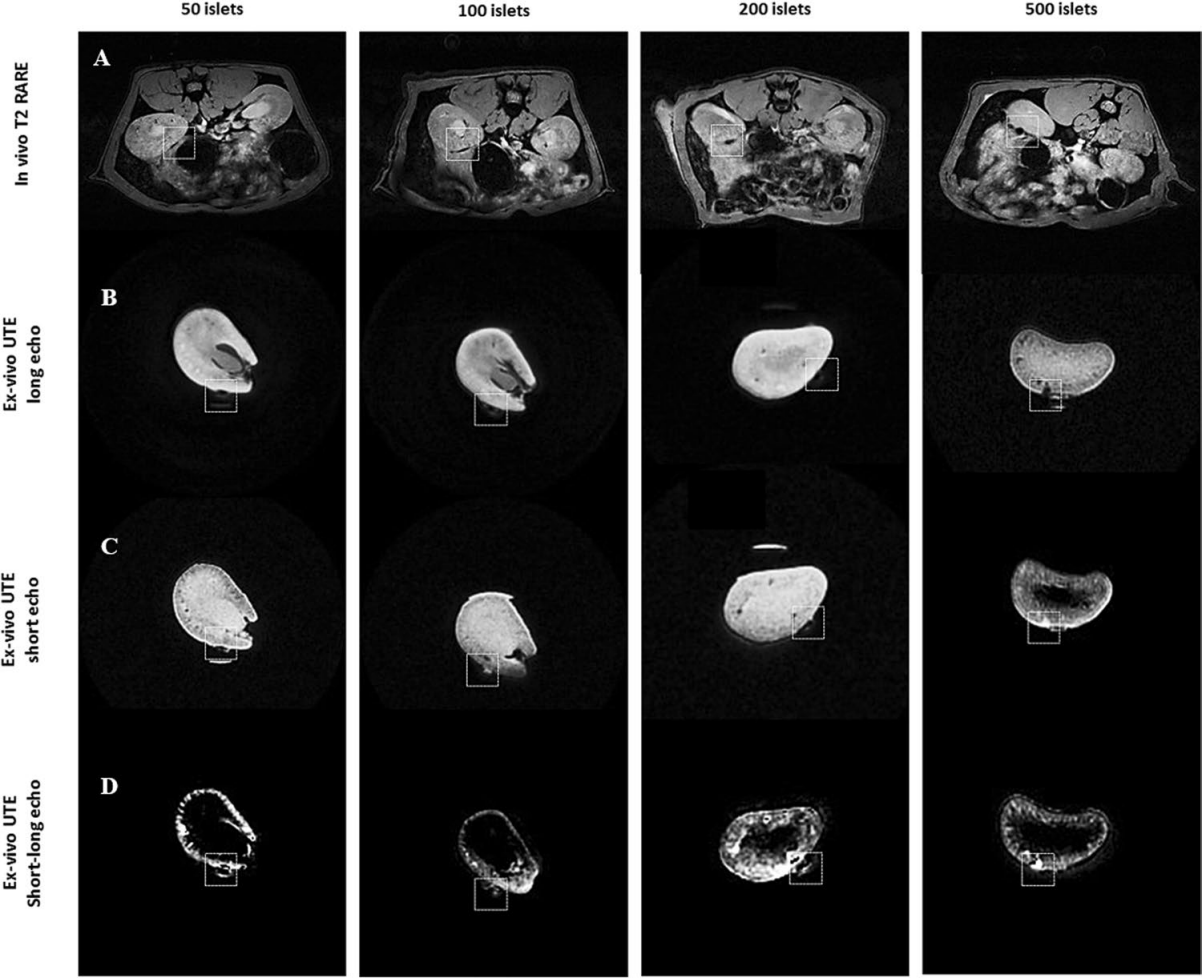
MR imaging was performed to detect labelled islets *in vivo* after sub-capsular kidney transplantation of 50–500 MLs-labelled pancreatic islets. Row (**A**) illustrates the *in vivo* MR images (2D-T2 weighted RARE sequence) Rows (**B**) to (**D**) are *ex vivo* MR images (UTE sequence) of the same kidneys as shown in row (**A**). Rows (**A**,**B**) indicate prominent hypointense region (indicated by white square) seen on the sub-capsular region after islets transplantation labelled with MLs. Row (**C**) indicate prominent hyperintense region (indicated by white square) seen on the sub-capsular region after islet transplantation labelled with MLs. Row (**D**) represent the subtraction of short (**B**) and long (**C**) TE from 3D UTE sequence.

The feasibility to non-invasively visualize transplanted islets and the minimal detectable cell numbers were investigated in healthy, non-diabetic rats by using *in vivo* MRI. We first implanted pancreatic islets (50–500 pancreatic islets), isolated from Lewis rats and labelled with MLs (50 µg Fe/ml for 24 hrs), under the left kidney capsule of non-diabetic (inbred) Lewis rats, which primarily shortens transverse relaxation times (*i*.*e*. T2 and T2*), leading to prominent signal decrease or “negative contrast” in MR images. Figure 4A shows a marked decrease in the signal intensity (white square) on T2*-weighted MR images of pancreatic islets labelled with MLs, for up to 45 days after implantation. To confirm these *in vivo* MR imaging results, we also performed *ex vivo* MRI of the left kidneys after surgical nephrectomy. *Ex vivo* MRI confirmed a marked decrease in signal intensity on T2*-weighted MR images at the implantation site in the left kidney corresponding to the different numbers of transplanted islets (Fig. 4B). A typical drawback of the T2- and T2*-weighted negative MR contrast is its poor specificity in particular when used to study areas with low background signal, such as the abdominal region^24^. In order to distinguish between the T2* signal coming from the MLs and intrinsic contrast, manual subtraction of short and long TE MRI using a 3D UTE pulse sequences was performed. Subtraction of short TE and long TE UTE data showed a noticeable individual hyper-intense region (Figure S4) in the left kidney. Similar results were also obtained from the subtraction of the segmented FLASH MRI pulse sequence with and without an inversion preparation (Fig. 4C,D), suggesting the presence of islets labelled with MLs.

**Figure 5:**
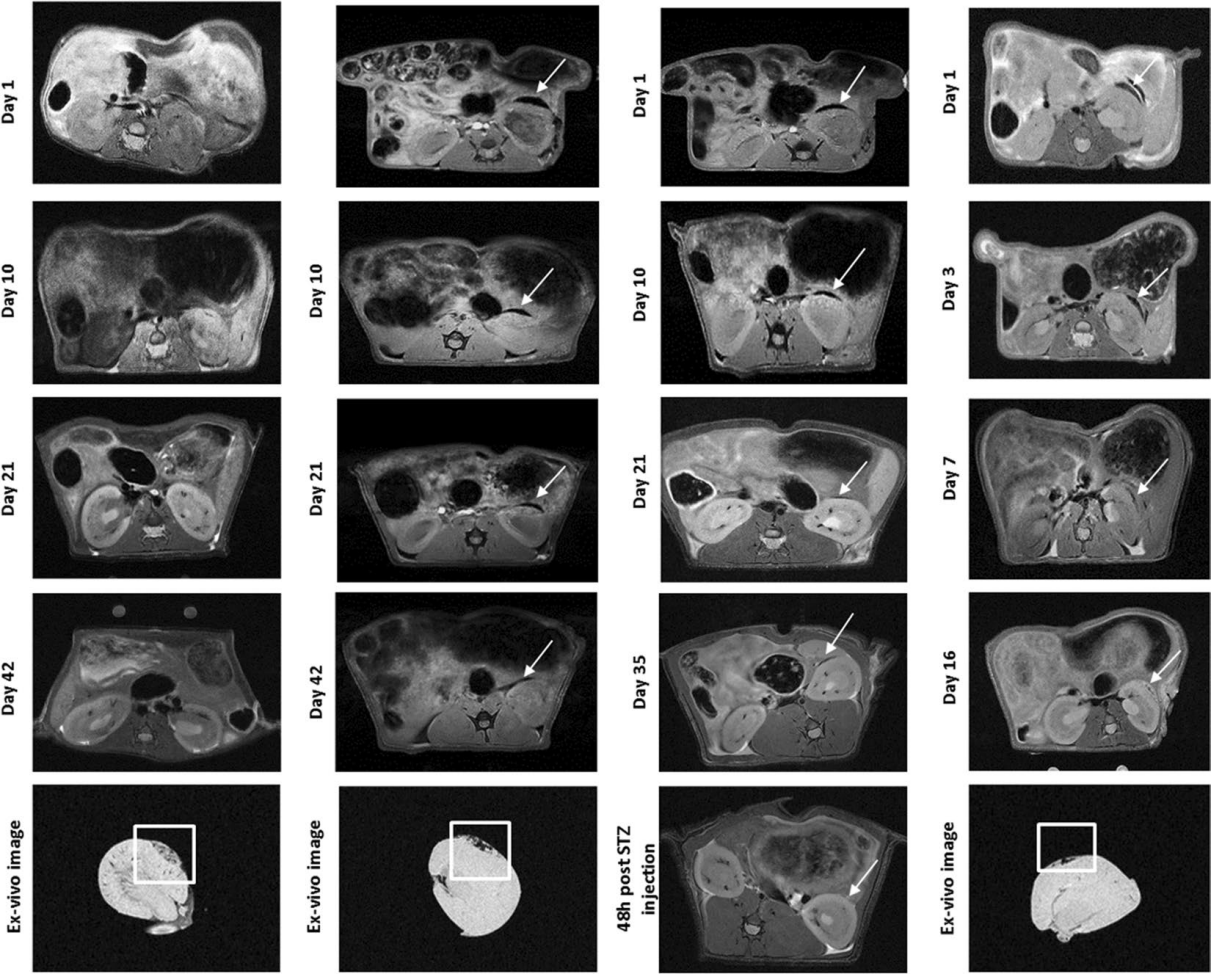
*In vivo* MRI (MSME sequence, echo = 8.41 ms) of MLs-labelled transplanted islets (50 µg Fe/ml) in diabetic rats during a 6 weeks longitudinal study. (**A**) STZ-induced inbred control (Lewis rats (n = 3), 3 weeks of age, 50 g, transplanted with 25 unlabelled pancreatic islets per g of BW), (**B**) STZ-diabetic inbred (Lewis rats (n = 3), 3 weeks of age, 50 g, transplanted with 25 MLs-labelled pancreatic islets per gram of BW), (**C**) Normoglycaemic (healthy (inbred) Lewis rats (n = 3), 50 g, transplanted with 25 MLs-labelled pancreatic islets per g of BW) and (**D**) STZ-diabetic outbred (Wistar rats (n = 3), 50 g, transplanted with 25 MLs-labelled pancreatic islets per g of BW).

Next, we investigated whether syngeneic islets labelled with MLs (25 pancreatic islets/g of body weight) could restore normoglycaemia in STZ-induced diabetic (inbred) Lewis rats. Both *in vivo* and *ex vivo* T2*-weighted MR images confirmed the absence of hypointense signal in the control graft (Fig. 5A) and the presence of MRI signal originating from labelled grafts in STZ-induced diabetic rats (Fig. 5B) with islet grafts being very discernible as a large hypointense spot (indicated by the white arrows) in the kidney capsule during the 6 week euglycaemic period. We obtained similar MR images in normoglycaemic rats (Fig. 5C), with ML-labelled islet grafts being very discernible as a large hypointense spot until 35 days post-transplantation, when an acute dose of STZ was administered. After 48hrs post STZ injection, it was no longer possible to identify any hypointense signal originating from labelled grafts. This indicates that upon islet death, MLs (released from islets and/or within dead cells) are removed by macrophages and dendritic cells. STZ-induced diabetic (outbred) Wistar rats transplanted with islets from Wistar rats (Fig. 5D) showed a prominent loss of ML-induced contrast from the graft site at day 7, confirmed by *ex vivo* MRI due to early rejection and recovery of the diabetic phenotype.

**Figure 6:**
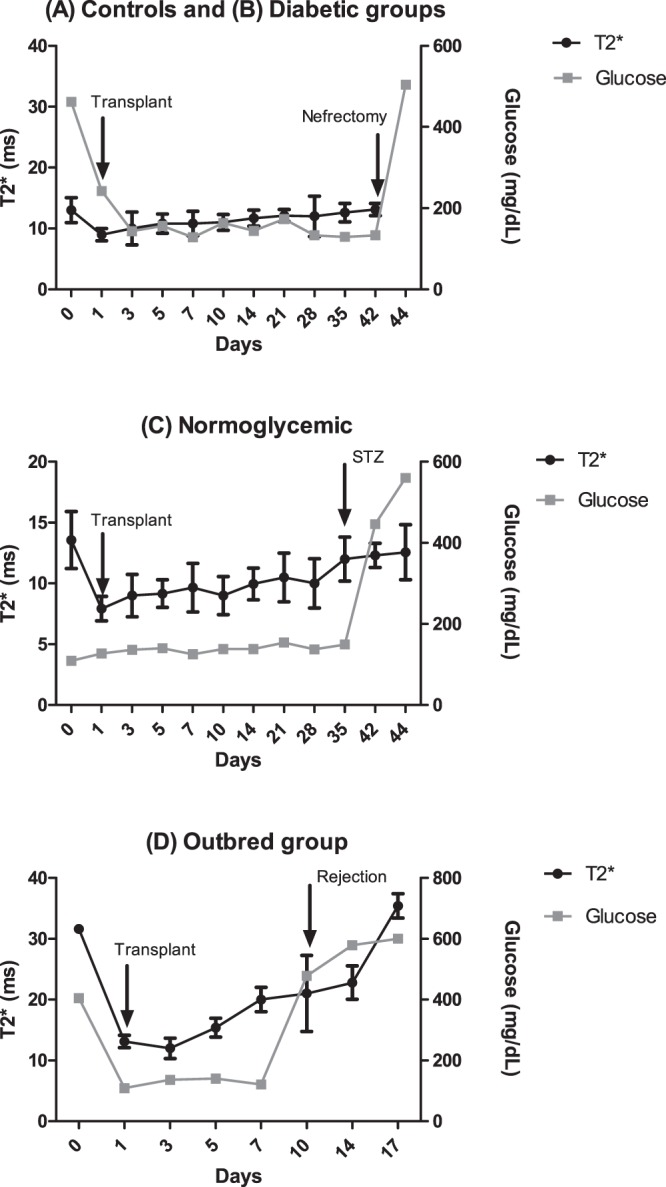
Blood glucose levels (mg/dL) and corresponding T2* values of recipient rats after transplantation with MLs-labelled islets. There was no significant difference in achieving normoglycaemia between **(A)** ‘control’ and **(B)** ‘diabetic’ animals (STZ-induced diabetic inbred Lewis rats transplanted with unlabelled/labelled MLs, respectively). All animals were nephrectomised after 6 weeks of normoglycaemia post transplantation and recovered hyperglycaemia in the following day. **(C)** ‘Normoglycaemic’ animals (non-diabetic inbred Lewis rats transplanted with labelled islets) were followed up for the duration of the experiment and injected with an acute dose of STZ at day 35 to prove that upon loss of graft animals become diabetic and MLs do not affect graft function *in vivo* pre-STZ injection. **(D)** ‘outbred’ animals (STZ-induced diabetic Wistar rats) were followed up for a period of 2 weeks showing a recovery of the hyperglycaemic status between day 7–16 correspondent with loss of MRI signal and suggesting graft rejection.

Blood glucose values were measured for all animals at the same days. The glycaemic profiles are also reflected in the T2* values (ms) measured over-time (Fig. 6). T2* values immediately after transplantation for (A) STZ-induced inbred controls and (B) STZ-diabetic inbred, from approximately 13 ± 2.0 ms to 9.0 ± 1 ms. These values stayed relatively constant until nephrectomy. For group (C) normoglycemic (healthy inbred), we found a similar reduction in T2* values immediately after transplantation (from 13.6 ± 2.3 ms to 7.9 ± 1 ms). These values remained stable until day 35 when an acute dose of STZ was administered and the islets die. This was reflected by an increase of T2* values to baseline values (12.6 ± 2.3 ms), which coincided with an increase of glucose levels, confirming the return to the diabetic status (>250 mg/dL). Finally, in group (D) STZ-diabetic outbred Wistar rats, we clearly observed that after day 3 post-transplantation the T2* values started to increase, reaching 20 ± 2 ms at day 7, while no change in glucose values were observed (constant around 120–140 mg glucose/dL). Only at day 10, an increase in glycemia was observed (glucose levels reaching 478 mg/dL), corresponding to a T2* values of 21 ± 6 ms. T2* values further increased until day 17 (35.4 ± 2 ms) with a simultaneous increase of blood glucose levels (maximum of 560 mg/dL).

**Figure 7:**
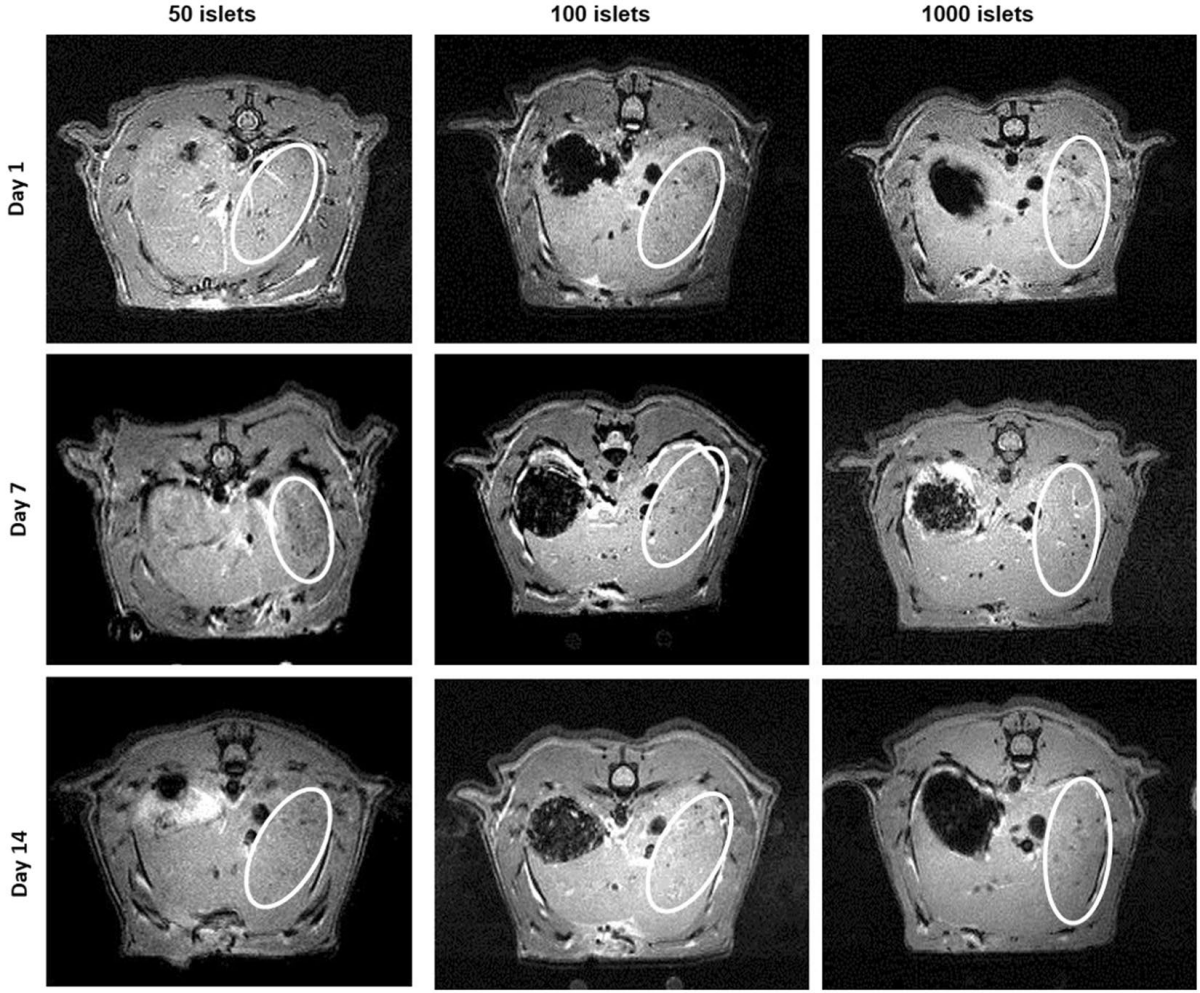
*In vivo* MRI (2D fast low angle shot (FLASH) sequence) of intrahepatic transplantation of MLs- labelled islets. A representative slice (0.5 mm) showing islets scattered throughout the liver. Pancreatic islets appear as hypointense spots on T2*-weighted images. Transplanted islet-related spots (surrounded by white circles) were detected by MRI in the liver 1 and 8 days after transplantation but only a minimal number of islet-related spots 14 days after transplantation.

We then transplanted separate sets of rats (*n* = 3 each) with 50, 100 and 1000 pancreatic islets labelled with MLs into the liver by infusion through the portal vein (Fig. 7). As expected, intrahepatic transplanted islets appeared as dark hypointense spots on the background of liver tissue, representing islet clusters. These clusters could be visualized with a detection threshold of at least 50 islets. Hypointense spots related to the labelled islets transplanted into the liver observed on day 1 post engraftment gradually disappeared and were almost absent on day 14, although some weak persisting hypointense spots can still be detected. Non-labelled islets transplanted intraportally could not be detected by MRI (Figure S5). A single infusion (n = 1) of 2000 MLs-labelled pancreatic islets was used as positive control (Figure S5).

### Histology

**Figure 8:**
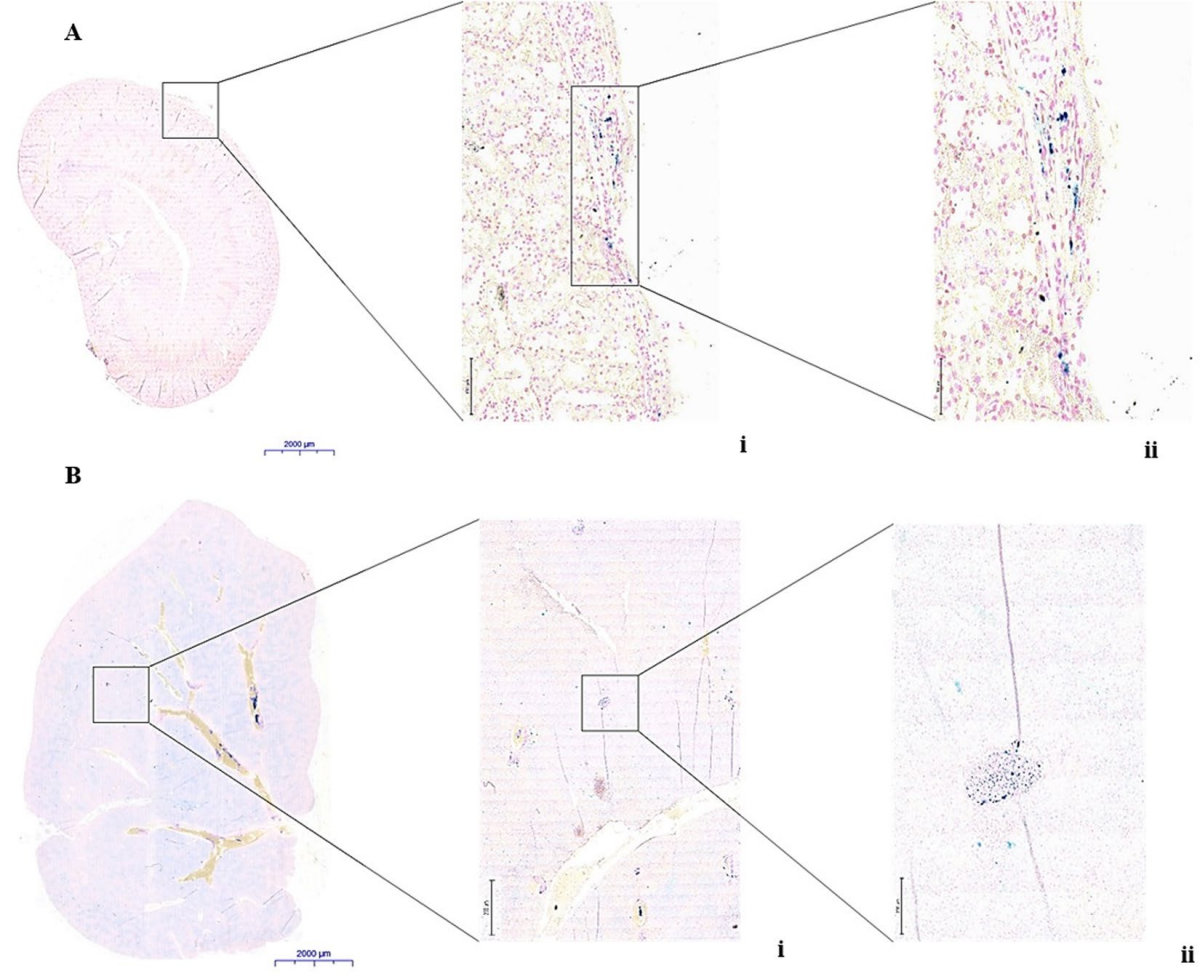
Prussian blue stain showing (**A**) MLs-labelled islet graft in the sub-capsular region of the kidney and (**B**) MLs-labelled pancreatic islets within the liver tissue of a portal vein infused rat. A (i) Scale bar = 100 µm. A (ii) Scale bar = 50 µm and B (i) Scale bar = 200 µm. B (ii) Scale bar = 50 µm.

To confirm the presence of iron labelled islet structures both in kidney and liver transplantation sites, tissue sections of both tissues were stained with Perl’s stain (Fig. 8). Iron-positive islets were observed in the subcapsular structure of all transplanted kidneys (Fig. 8A). Low intensities of iron particles were still detected 14 days after transplantation in cross-sections of the right liver lobes, confirming the presence of iron loaded islet-like structures in the liver parenchyma (Fig. 8B). Masson Trichrome stain also confirmed the presence of the islet graft in the left kidney capsule (Figure S6-A) of STZ-induced diabetic Lewis rats 45 days post- implantation. Only residual iron-positive structures were observed in the subcapsular structure of kidneys of the STZ-diabetic outbred Wistar rats 17 days post- transplantation (Figure S6-B). Small micro abscesses were observed at the estimated site of the graft (Figure S6-C) suggesting islet disintegration and clearance of the ML residues. Therefore, these experiments served as proof that labelling of pancreatic islets with MLs under the described conditions could reliably detect small islet numbers noninvasively by MRI.

## Discussion

Magnetoliposomes can easily be modified by changing the types of phospholipids and end groups that are incorporated in their phospholipid bilayer. Different lipid types and concentrations were previously tested, with 3.33% DSTAP MLs being chosen as the ideal lipid for cationic MLs (surface charge = 31.3 ± 7.3 mV), resulting in very high r_1_/r_2_ values (r_1_ = 15 ± 2 s^−1^/mM, r_2_ = 240 ± 8 s^−1^/mM) when compared to other SPIOs^28^. Anionic MLs, as used in the current study, contain a mixture of DMPC and DMPG, display a surface charge of −59.75 ± 5.12 mV, and a PDI of 0.36. This reduced the process of aggregation and makes the particles more stable for longer periods of time, which is a prerequisite for potential clinical use in the future. In addition, anionic MLs also display an increased r_2_ value (351 ± 3 s^−1^/mM) making them even more sensitive for *in vivo* longitudinal MR imaging. The relaxivity of the here used anionic MLs is about two times higher than that of other SPIO particles, e.g. 151 ± 6 s^−1^/mM for Resovist® at the same temperature and field strength^34^. In this study, optimal ML concentrations required for cell and islet labelling was defined as the minimum necessary for sufficient iron accumulation in the cells that would allow reliable MRI based detection without affecting islet or cell viability and function. Based on our *in vitro* labelling experiments, we adopted a standard labelling concentration for MLs of 50 µg Fe/ml medium for co-incubation over 24 hrs, which resulted in an average of 18.9 pg Fe/islet cell (assuming an average islet contains 1000 cells). These values are in agreement with previously reported levels for non-specific iron uptake by other cells when labelled with different types of SPIOs^22,35,36^.

Several groups have focused on using commercial and purposely developed SPIOs for the labelling of isolated pancreatic islets before transplantation, followed by non-invasive monitoring using MRI^22,37,38^. However, in comparison to this study where we obtained T2 values of 50.8 ± 16.2 msec (100 labelled islets) for a 50 µg Fe/ml of MLs labelling concentration for 24 hrs, others only achieved similar results after labelling with higher iron concentrations (*e*.*g*., 800 µg Fe/ml of ferumoxytol) and/or longer labelling times (*e*.*g*., for 48 hrs)^39^. In studies performed with Feridex®, it was possible to reduce the incubation times to overnight^13^ and even 1 hr^40^ by using either higher concentrations of iron oxide (200 µg Fe/ml) or by chemically conjugating the contrast agent directly onto the islet surface, respectively. Here, we propose a direct method for labelling of pancreatic islets without the utilization of transfection agents or further modifications to the islets themselves, at a very low iron oxide concentration (50 µg Fe/mL), which results in a high intracellular iron uptake and excellent T2/T2* MRI contrast. The lower iron concentration might ultimately result in better islet survival when compared to harsher labelling conditions. The higher MRI sensitivity of MLs can be explained by intracellular aggregation caused after partial degradation of the outer lipid layer in the lysosomal environment^32^.

We further assessed the feasibility of imaging transplanted islets labelled with MLs *in vivo* in healthy and diabetic Lewis (inbred) and Wistar (outbred) rats using two different sites of engraftment (the kidney capsule and the liver). A marked decrease in signal intensity on T2*-weighted MR images was detected at the implantation site of the left kidney when compared to controls, lasting up to 45 days post-transplantation. This is in accordance with other studies of renal subcapsular transplantation where islet grafts were easily detectable on T2*-weighted MR images as a pocket of signal loss disrupting the contour of the kidney at the transplantation site, which was stable for at least 30 days^13^. A typical drawback of the T2- and T2*-weighted negative contrast is its poor specificity. In addition, these images are not directly quantifiable. A number of approaches have been investigated to obtain positive contrast from SPIOs, including ultrashort echo time (UTE) MR imaging^40–42^. UTE MRI offers a relatively simple strategy by taking advantage of relatively high longitudinal relaxivities (r1) of SPIOs while reducing the contribution of predominant T2 and T2* contrast. With a very short echo time (<0.1 msec), UTE MRI captures signal enhancement from the T1 relaxation with little influence of signal decay from T2 and T2* relaxation, allowing for a positive contrast on T1-weighted UTE images^43^. *Ex vivo* UTE images indicate that MLs remained associated with the graft, showing a noticeable individual hyper-intense region in the left kidney capsule of transplanted animals. This was substantiated by subsequent histological analysis where iron-positive islets structures were observed.

It is also crucial for the outcome of transplantations that pancreatic islets labelled with a MRI probe retain their functionality, and moreover, their ability to secrete insulin. Caspase 3/7 activation assays and mRNA levels of ER stress mediators Chop, ATF4, Bip and XBP spliced did not show any increase after labelling with MLs for more than 24 hrs. This confirms that no significant activation of apoptotic pathways or ER stress markers in ML-labelled islets occurs under the conditions used for imaging. To ensure that labelling with MLs does not affect islet function, we transplanted islets labelled with MLs in STZ-treated Lewis rats and performed blood glucose measurements for a period of six weeks. From the day after transplantation, all animals became normoglycaemic for the duration of the experiment and there was no difference in achieving normoglycaemia between rats transplanted with labelled and unlabelled islets. On the other hand, after surgical nephrectomy at the end of the six weeks period of normoglycaemia, all animals became hyperglycaemic, confirming that the blood glucose regulation was only attributed to the transplanted islets, which was the case for both groups of rats transplanted with ML-labelled and unlabelled islets, respectively.

Interestingly, *in vivo* MRI of diabetic rats transplanted with outbred islets showed loss of islet-induced contrast between 7–16 days post-transplantation coinciding with a return of the hyperglycaemic status, indicating graft rejection. These results are in agreement with several studies where islet rejection has been reported during the first 10–14 days after transplantation^10,44–46^, suggesting that the observed loss of MRI contrast could be attributed to the effects of acute rejection of the xenogeneic transplants. Thus, we can infer that MRI of islets labelled with MLs allows not only localizing islets but also assessing their survival or destruction, which correlates with their function. This is especially relevant for clinical applications as the success of human islet transplantation highly depends on islet survival.

The renal sub-capsular region is a widely used site for PI transplantation in rodent models. However, this model is only suitable for short-term studies, due to the poor blood supply and the subsequent oxygen-poor microenvironment, which prevents longitudinal islet survival^47^. Finally, we applied ML-based islet labelling and imaging to islets transplanted at a hepatic site, which is currently the most frequently used protocol in clinic studies. We followed the fate of ML-labelled islets during the early post-transplantation period, believed to be crucial for graft survival. As expected, intrahepatically transplanted islets appeared as hypointense (dark) areas, representing islet clusters. These clusters could be clearly visualized if at least 50 ML-labelled pancreatic islets were engrafted (detection threshold). To our knowledge, the minimal amount of islets previously detected by *in vivo* MRI after pre-labelling with iron oxide based nanoparticles followed by intraportal transplantation was 300 islet equivalents (IEQ) at 7 T^40^ and 230 islets performed at 3 T^21^. The use of MLs improves the sensitivity of *in vivo* monitoring of engrafted pancreatic islets substantially. Using MRI-based monitoring of islets, it is not only possible to detect islet rejection early but also detect rejection of individual clusters of islets. These findings are in agreement with the model of Zacharovova *et al*. where almost all islet-associated contrast was eliminated from the liver after 14 days post transplantation^48^. However, we can still detect a few weak persisting hypointense spots on MRI, as also showed by Kriz *et al*.^49^. This could be due to the presence of iron in phagocytic cells around the transplant area. In contrast to blood glucose measurements, we were able to gain regional information on islet rejection, therefore we believe that this approach could potentially be translated into clinical practice for evaluating graft survival and for monitoring therapeutic intervention during graft rejection.

## Conclusion

In conclusion, labelling pancreatic islets with MLs was feasible in terms of MRI contrast requirements and safety. Labelling of islets with MLs did not interfere with their potential to correct the diabetic status in STZ-induced diabetic rats. Changes in MRI contrast allowed us to monitor immune rejection in the early post-transplantation period in an outbred rat model, coupled with a loss of islet functionality and recurrence of the diabetic phenotype. Furthermore, islets labelled with low concentrations of MLs enabled non-invasively monitoring of low numbers of transplanted islets (n ~ 50) by MRI. This approach could potentially be of interest for clinical application. It can be expected that the strong T2* MR contrast of MLs is sufficient to also allow the detection of transplanted islets at magnetic field strengths that are used in the clinic (1.5 or 3 T)^50^.

## Materials and Methods

### Synthesis and characterization of magnetoliposomes (MLs)

Anionic MLs were prepared as described in Supplementary Methods (S1). The integrity of the lipid bilayer of each ML sample was tested by determining the iron and phosphate content as previously described^33^. The particles were further characterized by Transmission Light Microscopy (TEM), Dynamic Light Scattering (DLS), zeta potential and relaxivity measurements. The parameters are described in detail in the Supplementary Methods (S2).

### Cell culture

INS-1E cells (originally obtained from Prof. C. Wollheim, Centre Medical Universitaire, Geneva, Switzerland) were maintained at 5% CO_2_ and 37 °C in RPMI 1640 medium supplemented with 10% heat inactivated foetal bovine serum, 1 M HEPES Buffer, 100 U/ml penicillin, 100 mg/ml streptomycin, 100 mM sodium-pyruvate and 10 mM β-mercaptoethanol (all from ThermoFisher Scientific (Invitrogen), Waltham, MA). Cells were kept in culture until passage 76 and split every week, with regular changes of medium every two days.

### Animals

We used 8 to 12-days-old Lewis (inbred) or Wistar (outbred) rats (Janvier, Saint-Berthevin, France) for pancreatic islet isolation. These islets were used for assessment of labelling, islet viability and function, and as donors for islet transplantation experiments. Lewis (inbred) or Wistar (outbred) rats of 22 days of age (50–60 g body weight, Janvier, Saint-Berthevin, France) were used as recipients for syngeneic and allogeneic islet transplantation experiments, accordingly to previous reports^47^. All principles of laboratory animal care were followed according to the Guide for the care and use of laboratory animals, Eighth edition (2011) and the latest European (Directive 2010/63/EU) and Belgian (Royal Decree of 29 May 2013) regulations on the protection of animals used for scientific purposes, and supervised by a qualified veterinarian. All animal experimental procedures were approved by the Ethics Committee of the KU LEUVEN (ECD number P076/2016).

### Pancreatic islet isolation

Pancreatic islets were freshly isolated by collagenase digestion^51^. The experimental conditions are described in detail in the Supplementary Methods (S3).

### MLs-cells interactions

A full experimental methodology on exposure conditions (S4), determining intracellular iron content (S5), Prussian Blue staining (S6), transmission electron microscopy (TEM) (S7), high content toxicity studies (S8), cell death assays (S9), caspase assays (S10), quantitative polymerase chain reaction (Q-PCR) for ER stress markers (S11) and Glucose Stimulated Insulin Secretion Assay (S12) are provided in the Supplementary Information.

### Islets transplantation experiments

Non-diabetic and STZ-induced diabetic (single intraperitoneal (i.p.) injection of 200 mg/kg STZ in citrate buffer) Lewis (inbred) and Wistar (outbred) rats were used as syngeneic and allogeneic islet recipients, respectively, for *in vivo* longitudinal follow up of islet location (MRI) and islet function after transplantation of pancreatic islets labelled with MLs. For the diabetic groups, only rats with blood glucose concentrations larger than 350 mg/dl were chosen as recipients, as previously described^51^.

To assess isograft function, blood glucose values were measured every other day with a precision blood glucose meter (Glucocard memory 2, Menarini, Florence, Italy). Full experimental details for the kidney capsule transplantation model (S13) and the intraportal liver transplantation model (S14) are provided in the Supplementary Methods.

### MRI experiments

All MRI measurements were performed using a 9.4 T Bruker Biospec small animal MR scanner (Bruker Biospin, Ettlingen, Germany; 20 cm horizontal bore) equipped with actively shielded gradients (600 mT m^−1^). *In vitro* and *in vivo* data were acquired using a quadrature radio-frequency resonator (transmit/receive, inner diameter 7.2 cm, Bruker Biospin).

Full experimental details on *in vitro* MRI of labelled islets (S15), *in vivo* MRI of transplanted islets in diabetic rats (S16), *ex vivo* MRI (S17) and image analysis (S18) are provided in the Supplementary Information.

### Histology

After fixation of isolated kidneys and performing *ex vivo* MRI, samples were embedded in paraffin and 5 µm thick sections were made. Paraffin sections were deparaffinised and dehydrated by passing through a graded alcohol series. To identify USPIO-containing islets, Masson Trichrome and Perl’s staining were performed. Images were acquired using a Mirax Desk (Carl Zeiss, Göttingen, Germany).

### Statistical analysis

Statistical analysis was performed using Graphpad Prism 6 software (Graphpad, La Jolla, CA). All values are expressed as means ± SEM of the means of the samples. The “n” numbers in the result section relates to the number of animals or samples used for the experiment. All collected data were analysed using Student’s T-test, except when indicated. P-values ≤ 0.05 were considered statistically significant.

### Data availability

All data generated or analysed during this study are included in this published article (and its Supplementary Information files).

## Electronic supplementary material

Supplementary Information
