# Supplementary material for: Magnetoliposomes as Contrast Agents for Longitudinal in vivo Assessment of Transplanted Pancreatic Islets in a Diabetic Rat Model
Source: Sci Rep. 2018 Jul 31;8:11487. doi: 10.1038/s41598-018-29136-9 (PMC6068133; doi:10.1038/s41598-018-29136-9)
Supplement: Supplementary file 1 — Supplementary Information [file 41598_2018_29136_MOESM1_ESM.docx]

**Supplementary Information**

**Magnetoliposomes AS CONTRAST AGENTS for LONGITUDINAL *in vivo* AsseSsment of transplanted pancreatic islets in a diabetic rat model.**

Rita Sofia Garcia Ribeiro^1^., Conny Gysemans^2^., João Paulo Monteiro Carvalho Mori Cunha^2^., Bella B. Manshian^1^., Daniel Jirak^3,4^., Jan Kriz^5^., Juan Gallo^6^, Manuel Bañobre-López^6^, Tom Struys^7^, Marcel De Cuyper^8^., Chantal Mathieu^2^., Stefaan J. Soenen^1^., Willy Gsell^1^., Uwe Himmelreich^1*^.

^1^ Biomedical MRI/ MoSAIC, Department of Imaging and Pathology, Biomedical Sciences Group, KU LEUVEN, Herestraat 49, 3000 Leuven, Belgium.

^2^ Clinical and Experimental Endocrinology, Department of Chronic Diseases, Metabolism and Ageing, KU LEUVEN, Herestraat 49, 3000 Leuven, Belgium.

^3^ MR Spectroscopy Unit, Institute for Clinical and Experimental Medicine (IKEM), Videnska 1958/9, 140 21, Prague, Czech Republic.

^4^ Department of Biophysics, Institute of Biophysics and Informatics, First Faculty of Medicine, Charles University, Salmovska 1, 120 00, Prague 2, Czech Republic.

^5^ Diabetes Center, Institute for Clinical and Experimental Medicine (IKEM), Videnska 1958/9, 140 21 Prague, Czech Republic.

^6^ Diagnostic Tools & Methods / Advanced (magnetic) Theranostic Nanostructures Lab, International Iberian Nanotechnology Laboratory (INL), Av. Mestre José Veiga s/n 4715-330 Braga, Portugal.

^7^ Lab of Histology, Biomedical Research Institute, Hasselt University, Campus Diepenbeek, Agoralaan, B3590, Diepenbeek, Belgium.

^8^ Laboratory of BioNanoColloids, Interdisciplinary Research Centre, KULAK/KU LEUVEN, Etienne Sabbelaan 53, 8500 Kortrijk, Belgium.

**^*^**Corresponding Author

E-mail: [Uwe.Himmelreich@kuleuven.be](mailto:Uwe.Himmelreich@kuleuven.be); Tel: +32 16 330925; Fax: +32 16 330901

**Materials and Methods**

**S1. Synthesis of magnetoliposomes (MLs)**

In short, sonicated vesicles that contain a mixture of 1,2-Dimyristoyl-sn-glycero-3-phosphorylcholine (DMPC) and 2-Dimyristoyl-sn-glycero-3-phosphorylglycerol sodium salt (DMPG) (both from Avanti Polar Lipids, Alabaster, Alabama) (9:1) in TES buffer (5 mM, pH 7.0) were mixed at room temperature with a lauric acid- stabilized water compatible magnetic fluid at a lipid/ Fe_3_O_4_ weight ratio of 1:5. Subsequently, the mixture was dialyzed for three days against TES buffer with regular buffer changes (at least 15 times). Separation of the resulting MLs from the excess vesicles was performed by high-gradient magnetophoresis.

**S2. Transmission Light Microscopy (TEM), Dynamic Light Scattering (DLS), zeta potentials and relaxivity measurements of MLs**

A JEOL 2100 transmission electron microscope (JEOL Ltd., Tokyo, Japan) working at 80 keV was used to image the nanostructures. Cryogen frozen samples were prepared in a FEI Vitrobot™ (ThermoFischer, Oregon, USA) under the following parameters: Sample volume, 7.5 µL; Blot time, 3 s; Wait time, 1 s; Drain time, 0 s; Blot force, -3; Blot total, 1. Lacey carbon coated, 300 mesh, copper grids (Ted Pella) were used for the samples. Hydrodynamic size and surface charge studies were performed on a Horiba nanoPartica SZ-100 (Horiba Ltd., Kyoto, Japan) directly in water solutions. In both cases, results are shown as the average of four independent measurements plus/minus the standard deviation. Measurements were performed directly in water at 37ºC in disposable cuvettes. For zeta potential measurements, carbon was the material of choice for the electrodes. For relaxivity measurements we used a 1.5 T MiniSpec relaxometer (Bruker BioSpin, Germany) with a saturation recovery sequence (t1_sr_mb), recycle delay 10s, 10 data points, first pulse separation 2 ms, final pulse separation 12 000 ms, delay sampling window 0.05 ms, sampling window 0.02 ms, 2 scans, and a Carr-Purcell-Meiboom-Gill sequence (t2_cp_mb), recycle delay 10 s, 1 ms interpulse delay, 4 scans, bandwidth 20 000 Hz, 500 - 2000 points. Samples were measured at 37°C.

**S3. Pancreatic Islet Isolation**

Briefly, the experiments were performed using 8 to 12 days-old Lewis or Wistar rats. Islets were obtained by dissecting the pancreases and placing them immediately in Hank’s medium. Appropriate measures were taken to minimize pain and discomfort for the rats, which were terminally anesthetised for the islet isolation. The islets were isolated by enzymatic digestion of the pancreas in 5 ml collagenase P solution (SERVA NB8, 1 mg/ml). After rinsing with Hank’s medium, the digest was centrifuged at 2000 g for 2 min. The supernatant was discarded. A volume of 10 ml of dextran (0.28 g/ml) was added to the residue. A three-layered dextran gradient was added (4 ml of 0.26 g/ml, 4.5 ml of 0.22 g/ml and 4 ml of 0.12 g/ml). The sample was centrifuged at 2000 g during 20 min. Islets were handpicked under a stereomicroscope (Nikon, Nikon SMZ 745T, Brussels, Belgium), and cultured in RPMI 1640 medium supplemented with 10% heat-inactivated foetal bovine serum (FBS), 100 U/ml penicillin, 100 mg/ml streptomycin, 2 mM GlutaMAX (all from ThermoFisher Scientific).

**S4. Cell labelling conditions**

INS-1 cells and pancreatic islets labelling experiments were performed by co-incubation of MLs in the culture medium using different labelling concentrations (0-100 µg Fe/ mL) for different incubation times (0-48hrs). Unlabelled cells/islets were always included as negative control in all the experiments.

**S5. Intracellular iron content**

Quantification of the intracellular iron content in the INS-1E cells/ pancreatic islets was performed with inductively coupled plasma optical emission spectrometry (ICP-OES, Varian 720ES, Santa Clara, CA, USA). Labelled INS-1E cells/ pancreatic islets, subjected for 24 hrs to co-incubation with medium containing MLs (10-50 µg Fe/ml), and were washed three times with PBS. Subsequently, INS-1E cells were trypsinised, the cell suspension was spun down (500 *g*) and counted. Cell pellets of 1 × 10^5^ INS-1E cells or 25 pancreatic islets were lysed with concentrated HCl (3.7%). Samples were further homogenized with distilled water. Standard solutions of 0.1, 0.5 and 1.0-ppm iron were measured before the first sample and after every 10^th^ sample.

**S6. Prussian blue staining**

To confirm uptake of MLs by INS-1E cells/ pancreatic islets, Prussian blue staining was performed. INS-1E cells/pancreatic islets, labelled for 24 hrs with different concentrations of MLs ranging from 10-50 µg Fe/ml medium, were washed three times with PBS. Labelled cells/ pancreatic islets were fixed in 4% paraformaldehyde (PFA) for 15 min at room temperature (RT). Two percent HCl (Vel Labs) and 2% potassium ferrocyanide (Sigma, St. Louis, MO) were mixed in equal volumes followed by 15 min incubation at room temperature. Samples were washed three times with PBS before light microscopy (Olympus, Münster, Germany).

**S7. Transmission Electron Microscopy (TEM) of completely pancreatic islets**

TEM analysis was performed to determine SPIOs distribution within pancreatic islets. Approximately 25 pancreatic islets labelled with MLs were fixed with 2% glutaraldehyde (Laborimpex, Brussels, Belgium) in 0.05M sodium cacodylate buffer (Aurion, Wageningen, The Netherlands). Samples were further prepared as previously described with minor modifications [1]. Briefly, following fixation, pancreatic islets were dehydrated in graded concentrations of acetone and embedded in araldite epoxy resin (Aurion, Wageningen, The Netherlands). Samples were cut in sections of 40-60 nm, using a Leica EM UC6 microtome (Leica Microsystems Belgium BVBA, Diegem, Belgium) and transferred to 50 mesh copper grids (Aurion, Wageningen, The Netherlands) coated with 0.7% formvar. TEM analysis was performed with a Philips EM208 S electron microscope (Philips, Eindhoven, The Netherlands) operating at 80 kV and provided with a Morada Soft Imaging System camera to acquire high resolution images of the samples. The images were processed digitally with the iTEM-FEI software (Olympus SIS, Münster, Germany).

**S8. High content image analysis for cell viability, mito ROS, LC3 and morphology**

High content (HC) imaging studies were conducted as previously described [2]. Cells were seeded in 96 well plates (Nunc, Belgium) at 1.5 × 10^3^ cells/mL in 100 µL culture media and allowed overnight incubation in a humidified atmosphere at 37 °C and 5% CO_2_. The following day cells were exposed to the NPs for 24 hrs in culture medium at 0, 5, 25, 50, 75, and 100 µg/ml concentrations. All experiments were performed in triplicate. Following the 24 hrs exposure period cells were washed twice with PBS and were immediately fixed with 4% paraformaldehyde (PFA) to prepare for LC3 and actin staining. Cells were then permeabilised for 10 minutes with Triton X-100 (0.3%) and blocked for 30 minutes with 10% serum-containing PBS, followed by room temperature incubation with primary murine anti-LC3 antibody (Cell Signalling Technologies, Belgium) or Acti-stain 488 phalloidin (TebuBio, Belgium). Anti-LC3 stained cells were then washed twice with PBS and further stained with AF488-coupled secondary goat anti-mouse antibody (Molecular Probes, Life Technologies Europe, BV, Belgium). All cells were finally washed twice with PBS and counterstained with 1:500 Hoescht (Thermo Fisher Scientific, Belgium) for 10 min in the dark at room temperature. For samples reserved for viability and mitochondrial ROS studies, live cells were incubated at 37 °C and 5% CO_2_ for 30 min with LIVE/DEAD® Fixable Green Dead Cell Stain Kit (Thermo Fisher Scientific, Belgium) and MitoTracker Red CMXRos (Molecular Probes, Life Technologies Europe, Belgium). Cells were then washed twice with PBS and counterstained with Hoechst. All samples were kept in PBS, in the dark at 4^o^C, until needed for analysis on the InCell analyser 2000 (GE Healthcare Europe GmbH, Belgium) microscopy instrument. Data processing was performed on the Investigator Tool 1.6.1 where cells were segmented and fluorescence intensities were determined in individual cells. Approximately 2000 cells were analysed per condition per replicate. Results were expressed as the mean + SEM of the untreated control values. Acquired data were analysed using one-way ANOVA. Dunnett’s post hoc test was applied to determine statistical significance of treated groups relative to the control group or between two treated groups under one condition.

**S9. Cell death assay**

The percentage of living, apoptotic and necrotic cells was assessed by microscopic cell counting after 4, 24 and 48 hrs of exposure to MLs (50 µg Fe/ml). INS-1E cells were plated in a 96-well plate and incubated for 15 min with propidium iodide (PI, 20 µg/ml, ThermoFisher Scientific (Invitrogen)) and Hoechst HO342 (4 µg/ml, ThermoFisher Scientific (Invitrogen)) at 37°C. At least 500 cells were evaluated for each experimental condition by two independent researchers, unaware of the sample’s identity, using an inverted fluorescent microscope (Nikon SMZ 745T, Brussels, Belgium).

**S10. Caspase 3/7 activity**

INS-1E cells were plated in a 96-well plate and exposed for different times (4, 24 and 48 hrs) to MLs (50 µg Fe/ml). Luminescence was measured on a Victor Wallac workstation (Perkin Elmer, Zaventem, Belgium), using the Caspase Glo 3/7 assay (Promega, Madison, WI), according to the manufacturer’s instructions.

**S11. Quantitative polymerase chain reaction (Q-PCR)**

Total RNA was extracted using the RNeasy® Micro Kit (Qiagen, Antwerp, Belgium) according to the manufacturer’s instructions. After priming with oligo dT, 0.5 mg RNA was converted to single stranded complementary DNA using Superscript II RT (Life Technologies, Ghent, Belgium) at 42°C for 80 min. mRNA levels of CCAAT/-enhancer-binding protein homologous protein (CHOP), Activating Transcription Factor 4 (ATF4), binding immunoglobulin protein (Bip) and of spliced X-box binding protein 1 (Xbp1s) were obtained by Q-PCR using a StepOne real-time PCR system (Applied Biosystems, Ghent, Belgium) and Fast SYBR Green Mastermix (Qiagen Antwerp, Belgium), based on the ∆∆Ct quantification method [3]. Values were normalized to the geometric mean of housekeeping genes 60S ribosomal protein L27 (RLP27), beta-actin and hypoxanthine-guanine phosphoribosyl transferase (HPRT), whose expression was not influenced by experimental conditions. Melting curve analysis confirmed primer specificities.

**S12. Glucose Stimulated Insulin Secretion Assay**

Glucose-stimulated insulin secretion assay was performed in whole rat islets after exposure for 24h, 48h and 72h to MLs (50µg Fe/mL). Islets were washed and equilibrated in glucose-free Krebs-Ringer bicarbonate HEPES Buffer (KRHB) containing 125 mM NaCl, 4.74 mM KCl, 1.2 mM KH_2_PO_4_H_2_O, 1.2 mM MgSO_4_.7H_2_O, 1 mMCaCl_2_.2H_2_O, 5 mM NaHCO_3_, 25 mM HEPES, and 0.1% BSA). The solution was then replaced with low glucose KRHB (3 mM) or high glucose KRHB (30 mM for islets), cells/islets were incubated for 1h at 37°C. Supernatant as well as remaining insulin content (extracted with acidic ethanol) was recuperated for insulin determination using an anti-mouse insulin ELISA kit (Mercodia, Uppsala, Sweden) according to the manufacturer’s instructions. Secreted insulin concentrations are expressed as the stimulation index, calculated as the ratio of stimulated (30 mM) to basal (3 mM) insulin release.

**S13. Kidney Capsule Transplantation Models**

For islet transplantation in the kidney’s subcapsular space, animals were anaesthetized with 1-2% isoflurane (mixed with 100% O_2_). The left kidney was exposed through a lumbar incision and capsulotomy was performed on the caudal outer layer of the left kidney. Different groups varying in islet numbers and glycaemic conditions were injected:

*Group (1)* Non-diabetic inbred Lewis rats (50, 100, 200, 500 pancreatic islets/rat, n=3 for each islet concentration) were used for optimization of MRI methods and testing detectability limits (number and time) of islets grafts.

*Group (2)* STZ-induced diabetic inbred Lewis rats (25 pancreatic islets/g of body weight, n=6). Long-term acceptance of the islet grafts was defined as persistence of normoglycaemia (random blood glucose below 200 mg/ dl) for more than 6 weeks post-transplantation. Three recipients were nephrectomised after 6 weeks of normoglycaemia post-transplantation (left kidney with iso-islet graft) to confirm that the grafts were responsible for the correction of the diabetic status.

*Group (3)* Non-diabetic inbred Lewis rats (25 pancreatic islets/g of body weight, n=3) were followed up for the duration of the experiment and injected with an acute dose of STZ at day 35 to prove that upon loss of graft, animals become diabetic and MLs do not affect graft function *in vivo* pre-STZ injection.

*Group (4)* STZ-induced diabetic outbred Wistar rats (25 pancreatic islets/g of body weight, n=3) were used to visualize islet rejection in the early post-transplantation period (10-16 days).

**S14. Intraportal Liver Transplantation model**

For liver transplantation through the portal vein of the liver, 50-2000 pancreatic islets were inserted into the liver of non-diabetic inbred Lewis rats using previously described methods [4]. Briefly, animals were anaesthetized with 1-2% isoflurane (mixed with 100% O_2_). The left hepatic branch of the portal vein was temporarily clamped, and islets were injected into the ileocaecal vein using a 27-G needle butterfly catheter (BD Science, United States), as previously described [5]. Twenty seconds after the injection of the islets, the vascular clamp was removed and the puncture site was surrounded with microfibrillar collagen hemostat (Avitene, Bard Canada, Inc., Mississauga, Ontario, Canada). Bleeding from the puncture was controlled using gentle pressure with a cotton swab.

**S15. *In vitro* MR imaging of labelled islets in phantoms**

T2* maps were acquired using a multi Gradient Echo (MGE) sequence (array of 14 echoes starting from 3.1ms and with regular echo spacing of 6ms, TR: 5000 ms, flip angle: 30 degrees, spectral bandwidth of 130 kHz, field of view (FOV): 70 x 70 mm, matrix: 256 x256 conferring an in-plane resolution of 273 μm^2^, single axial slice of 1mm thickness, acquisition time: 16 min). T2 maps were acquired using a multi-slice multi-echo (MSME) sequence (array of 20 echoes starting with the minimum echo time of 11.35ms and equally spaced increments of 11.35 ms, TR: 5000 ms, 4 averages, FOV: 60 x 60 mm, matrix: 512 x 512 conferring an in-plane resolution of 117 μm^2^, five non-contiguous slices of 1mm thickness with 1mm gap, acquisition time: 1hr54min). T1 map were acquired using an inversion prepared spin-echo (IR-SE) sequence (TE: 5.67 ms, TR: 18000 ms, array of 12 inversion times starting from 69 ms and equally spaced by 500ms, using the same geometry as for the T2 map acquisition but with a matrix of 256 x 256 conferring an in-plane resolution of 234 μm^2^, acquisition time: 6hr12min).

**S16. *In vivo* MR imaging of transplanted islets in rats**

For the kidney scanning, we used a 2D-T2 weighted RARE sequence (effective TE: 25.18 ms, TR: 1200 ms, RARE factor: 8, 10 averages, FOV: 30 x 60 mm, matrix: 256 x 512, 2 slice packages of 7 axial slices of 1 mm thickness with 1 mm gap placed in an interleaved fashion providing coverage of 14mm in z dimension with no gap, acquisition time: 12 min 48s) followed by a MSME (array of 16 echoes starting with the minimum echo time of 8.41 ms and equally spaced, TR: 3000 ms, FOV: 60 x 60 mm, matrix: 392 x 392, 13 axial slices of 1mm thickness and 1 mm gap, acquisition time: 13min 06s) and a MGE (array of 20 echoes starting from 2.31 ms and with regular echo spacing of 2.8 ms, TR: 1200 ms, flip angle: 30 degrees, spectral bandwidth of 200 kHz, field of view (FOV): 50 x 60 mm, matrix: 392 x392, 10 axial slices of 1mm thickness with 1mm gap, acquisition time: 10min 38s). For the liver grafting study, animals were scanned using the following protocol: 2D fast low angle shot (FLASH) sequence (TE: 2.32 ms, TR: 600 ms, flip angle: 30 degrees, 4 averages, spectral bandwidth: 75kHz, FOV: 70 x 70 mm, matrix: 256 x 256, 42 interlaced contiguous axial slices of 0.6mm thickness, acquisition time: 7min 41s). MGE (array of 16 echoes starting from 1.82 ms and with regular echo spacing of 2.1 ms, TR: 1500 ms, flip angle: 30 degrees, spectral bandwidth of 200 kHz, field of view (FOV): 70 x 60 mm, matrix: 392 x 392 conferring an in-plane resolution of 178 μm^2^, 2 slice packages of 10 axial slices of 1.25 mm thickness with 1.25 mm gap placed in an interleaved fashion to cover the full liver with no gap, acquisition time: 6 min 33s). MSME (array of 8 echoes starting with the minimum echo time of 8.6 ms and equally spaced, TR: 2000 ms, 2 averages, FOV: 70 x 60 mm, matrix: 128 x 192, using the same 2 slice packages described previously for the MGE, acquisition time: 9min 36s).

**S17. Ex vivo MRI validation**

In order to confirm the results from *in vivo* MRI scans, we performed *ex vivo* high resolution MRI scans. Left kidneys were isolated 6 weeks after islet transplantation and fixed overnight in 10% neutral buffered formalin. Before scanning, samples were placed in a 2ml syringe (Braun Melsungen AG, Germany) filled with perfluoropolyether (Fomblin®, Solvay Solexis, Thorofare, New Jersey). We acquired a 3D ultra-short echo time (UTE) sequence (TR: 6.663 ms, 10 averages, flip angle: 15 degrees, spectral bandwidth: 100 kHz, FOV: 30 x 30 x 30 mm, matrix: 192 x 192 x 192, acquisition time: 2hrs9 min). We then acquired a T2* weighted 3D gradient echo (TE: 2.53 ms, TR: 350 ms, flip angle: 30 degrees, spectral bandwidth of 200 kHz, FOV: 31 x 30 x 30 mm, matrix: 256 x 256 x 256, acquisition time: 4hrs47min). In a similar way to the strategy used with UTE but using the T1 properties of the MLs and the surrounding tissues, we acquired a segmented flash with and without inversion preparation also known as magnetization prepared rapid gradient echo (MPRAGE) with the following parameters: acquisition time: TE/TR: 4.13/12.06ms, 21 segments, flip angle: 20 degrees, 10 averages, FOV: 30 x 52 x 36 mm, matrix: 512 x 512 x 120, inversion time: 100 ms, acquisition time: 2hr49min.

**S18. Image Analysis**

Images were processed using the Bruker Biospin software Paravision 5.1. T1, T2- and T2*-maps were reconstructed in Paravision 5.1, through a pixel wise mono-exponential fit. For the phantom experiment different circular regions of interest (ROIs) of 6mm^2^ each, corresponding to the different labelling conditions in the phantom, were appointed to extract T1, T2 and T2* relaxation time. For the kidney samples in order to distinguish between the T2* signal coming from the MLs and the background, manual subtraction of short and long TE from 3D UTE sequences was performed using Image J software (NIH, USA) [6, 7].

**References**

1. Struys, T., Kektar-Atre, A. Magnetic resonance imaging of human dental pulp stem cells in vitro and in vivo. *Cell Transplant*. **22**, 1813–29 (2013).

2. Manshian, B.B., Pokhrel, S., Himmelreich, U., Tämm, K., Sikk, L., Fernández, A., *et al*. In Silico Design of Optimal Dissolution Kinetics of Fe-Doped ZnO Nanoparticles Results in Cancer-Specific Toxicity in a Preclinical Rodent Model, *Adv. Healthc. Mater*. **6**, 10.1002/adhm.201601379 (2017).

3. Schmittgen, T., Livak, K.J. Analyzing real-time PCR data by the comparative CT method. *Nature Protocols*. **3**, 1101 – 8 (2008).

4. Yonekawa, Y., Okitsu, T., Wake, K., Iwanaga, Y., Noguchi, H., Nagata, H., *et al*. A new mouse model for intraportal islet transplantation with limited hepatic lobe as a graft site. *Transplantation*. **82**, 712-5 (2006).

5. Kriz, J., Jirak, D., White, D., Foster, P. Magnetic resonance imaging of pancreatic islets transplanted into the right liver lobes of diabetic mice. *Transplat. Proc*. **40**, 444-8 (2008).

6. Wang, L., Zhong, W., Qian, W., Huang, J., Cao, Z., Yu, Q., *et al.* Ultrashort echo time (UTE) imaging of receptor target magnetic iron oxide nanoparticles in mouse tumor models. *J. Magn. Reson. Imaging*. **40**, 1071-81 (2014).

7. Schneider, A.C., Rasband, S.W., Eliceiri, K.W. NIH Image to Image J: 25 years of image analysis. *Nature Methods*. **9**, 671-5 (2012).

8. YX., Wang. Superparamagnetic iron oxide based MRI contrast agents: Current status of clinical application. *Quant Imaging Med Surg*. **1**, 35-40 (2011).

9. Ribeiro R, Ketkar-Atre A, Yin T, Louchami K, Struys T, Lambrichts I, *et al*. Improved labeling of pancreatic islets for longitudinal monitoring by MRI using cationic magnetoliposomes. *J. Pers. Med.* **8**, 12, doi:10.3390/jpm8010012 (2018).

**Results and Discussion**

**Supplementary Table 1:** Physicochemical properties of superparamagnetic iron oxide MR contrast agents in comparison to the ones (anionic MLs) used in this study.

| Name | Coating | Hydrodynamic Size (nm) | Surface Charge (mV) | *r*_1_ ^§^  (s^−1^/mM) | *r*_2_ ^§^ (s^−1^/mM) |
| --- | --- | --- | --- | --- | --- |
| Resovist | Carboxydextran | 45–62 nm | −4.4 ± 2.59 | 25 ± 3 | 151 ± 6 ^(8)^ |
| Cationic MLs | 3,33% DSTAP | 40 nm | + 31.3 ± 7.3 | 15 ± 2 | 240 ± 8 ^(9)^ |
| Anionic MLs | DMPC: DMPG | 53.5 ± 0.3 nm | - 59.75 ± 5.12 | 15,71 ± 0.04 | 351 ± 3 |

§ 1.5 T, 37 °C.

**Supplementary Table 2:** MR relaxation time assessment of pancreatic islets labelled *in vitro* with MLs: T1, T2 and T2*-values of an agar phantom containing 100 pancreatic islets pre-labelled with different concentrations of MLs (0, 10, 25 and 50 µg Fe/mL) distributed in 200 µL agar. Results are expressed as the mean ± standard deviation (SD) of ROIs placed on each sample (6 mm^2^).

| Fe/mL (µg/mL) | T1 (ms) | T2 (ms) | T2* (ms) |
| --- | --- | --- | --- |
| a) 0 | 2470±24.5 | 73.0±13.2 | 39.7±12.5 |
| b) 10 | 2450±16.8 | 72.4±6.7 | 22.8±7.0 |
| c) 25 | 2470±20.7 | 52.5±5.6 | 19.0±10.9 |
| d) 50 | 2410±74.7 | 50.8±16.2 | 14.5±3.5 |

**
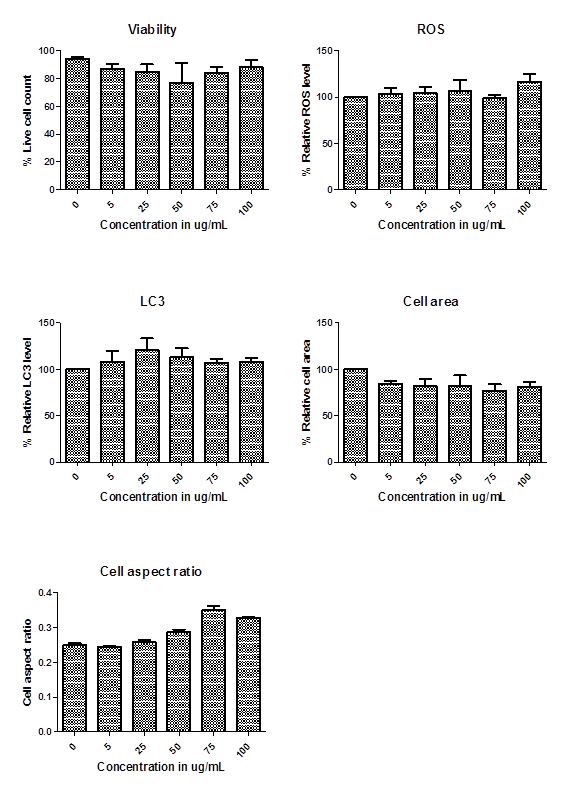
**

**Supplementary Figure 1:** High content image analysis for cell viability, mito ROS, LC3 and morphology (cell area and cell aspect ratio) for INS-1E cells labeled with increasing concentrations of MLs for 24 hrs. There are no statistical significance of treated groups relative to the control group or between two treated groups under one condition. Data are presented as mean ± SEM (One-way ANOVA).


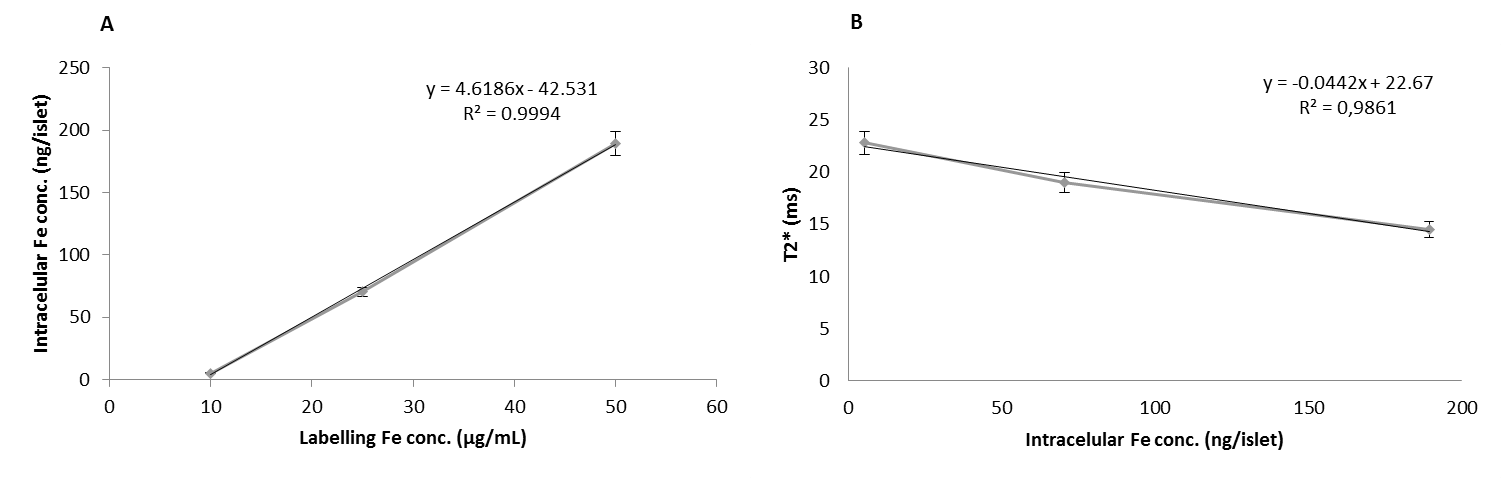


**A**

**B**

**Supplementary Figure 2:** Correlation between ML uptake and change in T2/T2* relaxivity in labelled islets. (**A**) ICP-EOS measurements of 100 IEQ labelled with increasing (0-50 µg Fe/mL) concentrations of MLs and (**B**) T2* values of agar phantoms containing 100 islets labelled with increasing (0-50 µg Fe/mL) concentrations of MLs distributed in 200 µL agar. A direct correlation between T2* values and intracellular Fe concentration was seen (r^2^= 0.9861). Iron uptake at a labelling concentration of 50 µg Fe/mL resulted in 189 ± 0.84 ng Fe/islet with a T2* relaxation time of 14.5 + 35 ms.

**Supplementary Figure 3:** Glucose stimulated insulin secretion in whole pancreatic islets (n=20) after 24, 48 and 72h of exposure to MLs. There was no significant difference between labelled islets vs. unlabelled islets. Data are presented as mean + SEM (Two-Way ANOVA).


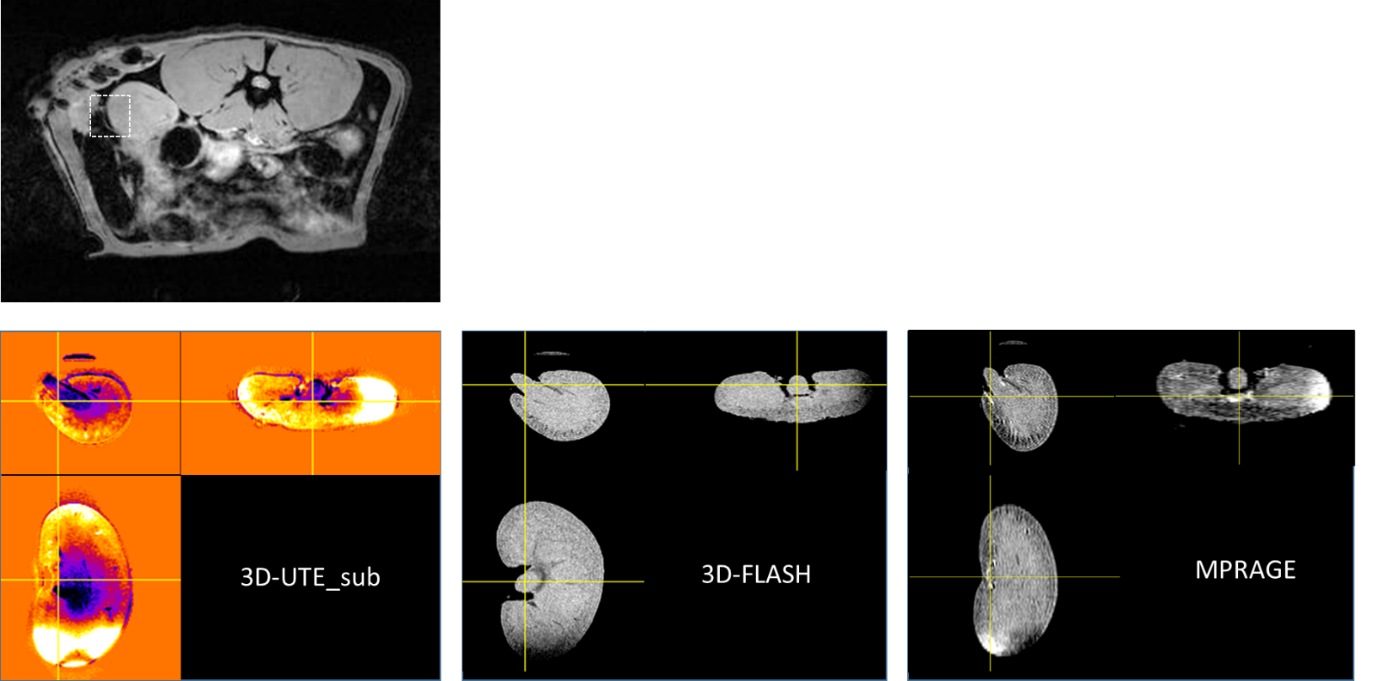


**Supplementary Figure 4:** Representative image of the **s**ubtraction of short and long TE from 3D UTE followed by its corresponding segmented FLASH and MRPAGE sequences for 200 MLs-labelled islets transplanted under the left kidney capsule. An individual hyper-intense region is noticeable in the kidney capsule suggesting the presence MLs-labeled islets.


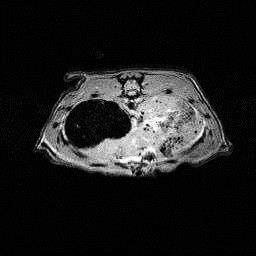


**Control**


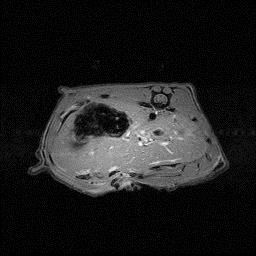


**2000 islets**

**Day 1**

**Supplementary Figure 5:** *In vivo* MRI of intrahepatic transplantation of (A) unlabeled islets and (B) 2000 MLs-labeled islets. A representative slice (0.5 mm) showing islets scattered throughout the liver. Pancreatic islets appear as hypointense spots on T2*-weighted images (transplanted region delineated by white circle).


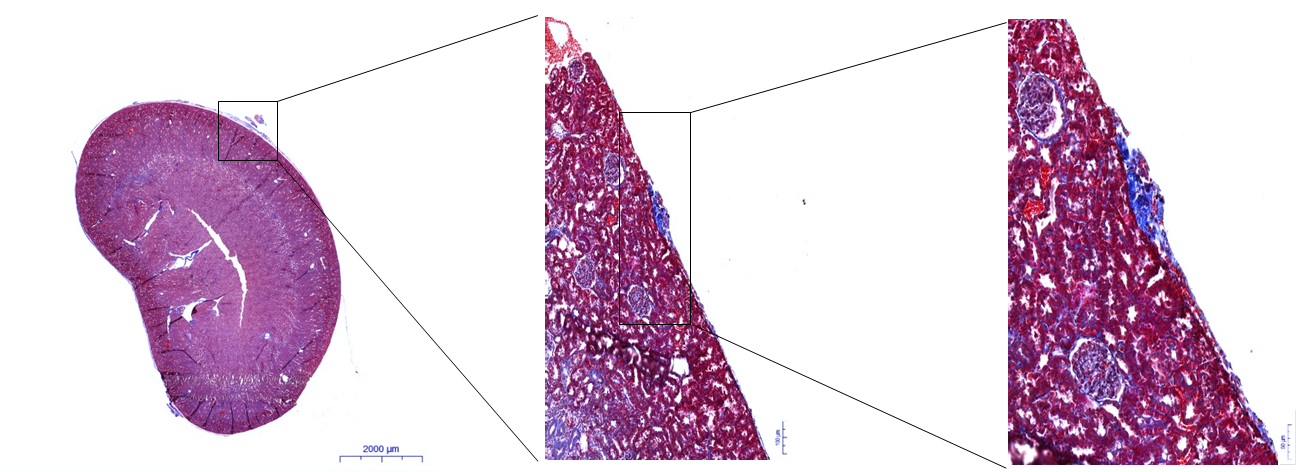


**i**

**ii**

**iii**


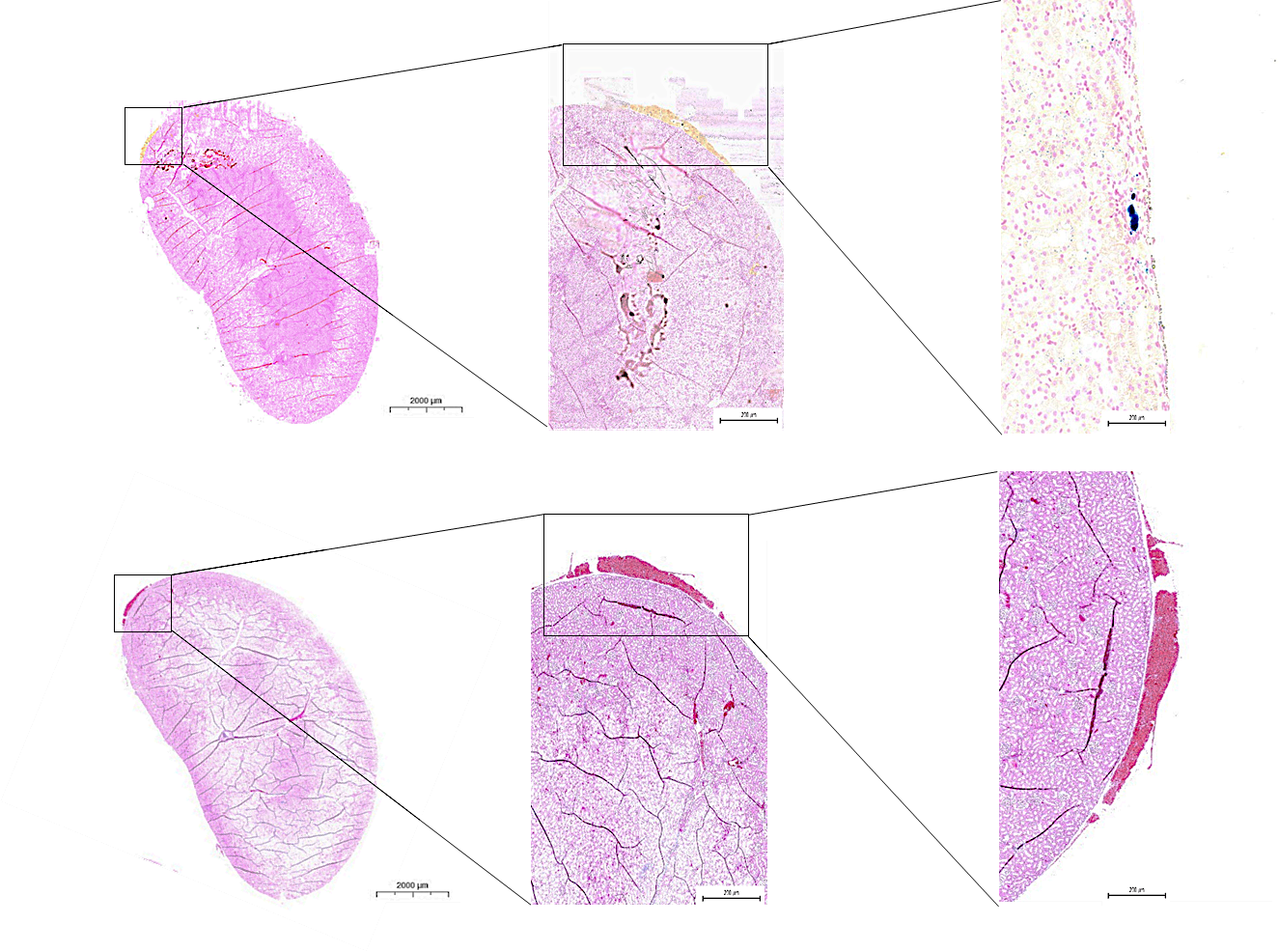


**B**

**C**

**A**


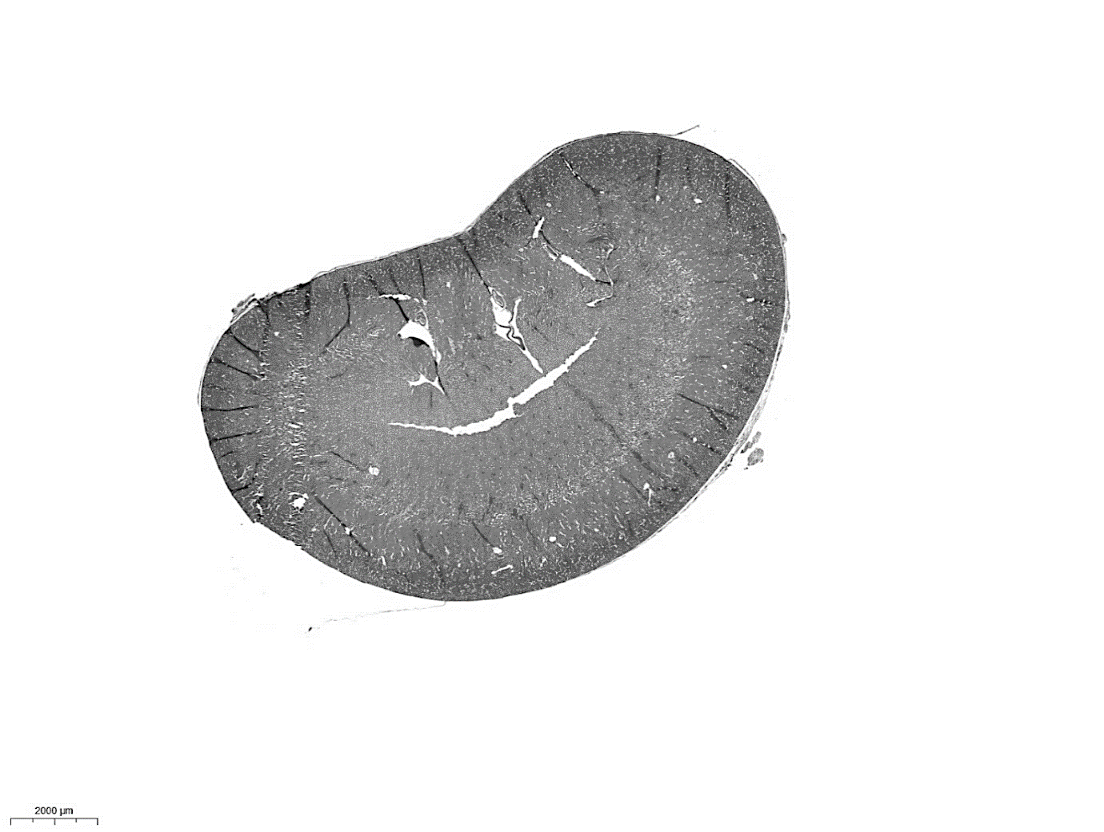


**ii**

**iii**

**i**

**
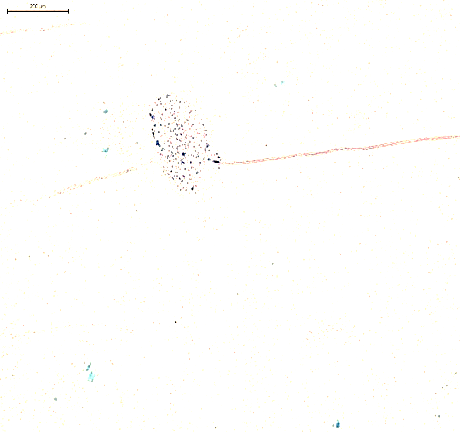
**

**iii**

**ii**

**i**

**iii**

**Supplementary Figure 6: (A)** Masson Trichrome stain showing MLs-labelled islet graft in the sub-capsular region of the kidney. **B)** Perl’s stain showing only residual iron-positive structures in the subcapsular structure of kidneys of the STZ-diabetic outbred Wistar rats 17 days post- transplantation. **(C)** Masson Trichrome stain showing the presence of a small micro abscess at the islet transplant graft site, suggesting islet disintegration and clearance of the ML residues. (i) Scale bar = 2000 µm. (ii) Scale bar = 100 µm and (iii) Scale bar = 50 µm.
